# You Don’t See What I See: Individual Differences in the Perception of Meaning from Visual Stimuli

**DOI:** 10.1371/journal.pone.0150615

**Published:** 2016-03-08

**Authors:** Timea R. Partos, Simon J. Cropper, David Rawlings

**Affiliations:** Melbourne School of Psychological Sciences, University of Melbourne, Victoria, 3010, Australia; Ecole Polytechnique Federale de Lausanne, SWITZERLAND

## Abstract

Everyone has their own unique version of the visual world and there has been growing interest in understanding the way that personality shapes one’s perception. Here, we investigated meaningful visual experiences in relation to the personality dimension of schizotypy. In a novel approach to this issue, a non-clinical sample of subjects (total n = 197) were presented with calibrated images of scenes, cartoons and faces of varying visibility embedded in noise; the spatial properties of the images were constructed to mimic the natural statistics of the environment. In two experiments, subjects were required to indicate what they saw in a large number of unique images, both with and without actual meaningful structure. The first experiment employed an open-ended response paradigm and used a variety of different images in noise; the second experiment only presented a series of faces embedded in noise, and required a forced-choice response from the subjects. The results in all conditions indicated that a high positive schizotypy score was associated with an increased tendency to perceive complex meaning in images comprised purely of random visual noise. Individuals high in positive schizotypy seemed to be employing a looser criterion (response bias) to determine what constituted a ‘meaningful’ image, while also being significantly less sensitive at the task than those low in positive schizotypy. Our results suggest that differences in perceptual performance for individuals high in positive schizotypy are not related to increased suggestibility or susceptibility to instruction, as had previously been suggested. Instead, the observed reductions in sensitivity along with increased response bias toward seeing something that is not there, indirectly implicated subtle neurophysiological differences associated with the personality dimension of schizotypy, that are theoretically pertinent to the continuum of schizophrenia and hallucination-proneness.

## Introduction

Like all human abilities and traits, there are individual differences in visual perception. Anecdotally, these can lead to seeing different things in a cloud-filled sky and appreciating particular forms of abstract art [1]. Experimentally, these variations range from slight, and perhaps random, fluctuations in performance across individuals [2–5], to considerable dissimilarities that can be reliably traced to broader group differences in, for example, gender [6], personality [7], culture [8], motivation [9], and the spectrum of psychosis [10, 11]. In the work described here we use the commonplace observation of meaningful images in the clouds to construct stimuli that allow us to measure individual differences in the inclination to see something in a degraded, noisy stimulus when nothing is actually there.

### Individual differences in perception

The visual system is predisposed to extract meaning, often from stimuli containing substantial amounts of uncertainty; indeed one could argue that is its primary role. The meaning that is imposed can depend on our internal templates of prototypical stimuli [12, 13] our expectations and learned probabilities about the visual environment [9, 14–20], contextual cues or prior visual input [21, 22] and on random neural fluctuations in functionally relevant areas of cortex [23–26]. Each of these processes are also subject to influence by other factors such as the personality (or factors which influence personality) of the individual.

For instance, when subjects were presented with a sequence of line-drawings, starting with an extremely degraded image that gradually became less degraded as the sequence progressed, and asked to respond once they could identify the image, those who responded earlier in the sequence, although less accurately, were those with stronger beliefs in paranormal phenomena [27]. Stronger belief in the paranormal is also associated with a greater bias toward seeing an illusory face during a signal-detection task that used real-world photographs, some of which contained “artifact faces” [28], the tendency to attribute intention and animacy to sequences of random motion [29], and to ascribe meaning to random sequences of everyday events [30]. Paranormal ‘believers’ also show a greater likelihood to see a face in a jumbled non-face, and a word in a non-word compared to ‘skeptics’ [31]. Overall, the results imply an influence of the internal state of the system (in this case a particular belief system) on the outcome of the process of construction of meaning, with a role for both of expectations [9, 14–20] and prototypical templates [12, 13].

### Schizotypy and perception

From a more generalised perspective, psychometric scales of personality have long been thought to influence aspects of perception which, in turn, influences behaviour [32]. Belief in extra-sensory perception and paranormal phenomena, and high scores on measures of psychoticism and magical thinking can be contextualised in terms of the personality dimension of schizotypy [31, 33]. The concept of schizotypy has often been employed within a dimensional approach to psychosis, whereby psychopathological symptoms are thought to form a quantitative continuum with normal, healthy traits [34–36]. The presence of schizotypal traits, even at relatively high levels, does not imply impaired functioning [37], but may be indicative of risk for psychosis-spectrum disorders [38, 39]. The normal personality traits comprising schizotypy typically cluster mainly into three subtypes: *positive-psychotic*, *positive-disorganized*, and *negative*, with a fourth subtype becoming increasingly, although not universally, recognized *impulsive/ antisocial* [40, 41]. Of particular interest for the present research is what has been termed the *positive-psychotic* subtype, which encompasses distorted or intense subjective sensory experiences and minor manifestations of delusional beliefs. This dimension also correlates well to various measures of creativity, contributing to the concept of the ‘healthy schizotype’ with which we concur [42–44], and we stress that in no way does any given schizotypy score imply a clinical diagnosis.

Experimentally, when observers were presented with a random array of white dots on a black background and (misleadingly) instructed that the dots sometimes show something meaningful, individuals who score higher on questionnaire measures of psychoticism, neuroticism, and hallucination-proneness are more likely to report perceiving meaningful images of a complex nature in the dots [45]. Such complex false alarms on this *Random Dots Task* (*RDT*) have also been associated with belief in extra-sensory perception [46], magical thinking and positive schizotypy [47]. Individuals high in positive schizotypy also see more words in a dynamic string of non-word strings than low-scoring individuals [48], mirroring the result from similar work with paranormal believers [31]. This effect is strengthened when the frequency of real words is increased [18], and is mediated by expectation, even when that expectation is not met [16]. These data are consistent with perceptual biases that predispose those high in positive-psychotic traits to have measurable false perceptions.

### Hallucination as a visual false-alarm

From a stimulus-based perspective, the strengths of visual illusions are mediated by various aspects of individual difference and clinical diagnosis [49–51], and there is evidence that both illusions and false-perceptions (or hallucinations) arise, at least in part, from the same mechanisms as ‘veridical’ perception. For instance, changes in local cortical blood flow are similar when an individual is looking at either illusory or ‘real’ contours [52], studies of the McCullough Effect [53] suggest the illusory colours inherent in the illusion are mediated through the same mechanism as the percept of the coloured adapting stimulus [54], and there is mounting evidence for common mechanisms mediating hallucinations and shared perception [55, 56].

The results outlined above suggest that illusions and hallucinations can be conceptualised as “visual false alarms”; an approach that fits within a probabilistic framework of vision and brain function [15, 17, 57], and is consistent with the tone of the argument characteristic of the literature. The overall aim of this work is to use the visual false alarm in this context to examine how personality interacts with perception.

### The current study

From a task-driven perspective, while much previous work used artificial stimuli and subjective judgements open to suggestibility [58, 59], the two experiments presented here outline the development and use of a more ecologically valid and controlled visual stimulus in a signal-detection task paradigm, allowing us to more confidently attribute performance to perceptual differences between individuals (see also [28, 31, 48]).

From a stimulus-driven perspective, we suggest that it is common to see apparently meaningful images in clouds because of their particular spatial structure, where the power in a given spatial frequency-band has an approximately reciprocal relationship to frequency (1/ *f*, where *f* is the spatial frequency), and the fractal characteristic of self-similarity across scale [60]. These structural visual properties stimulate the system relatively evenly across its range of sensitivity at early visual stages [61], and can be considered to create an overall increase in ‘noise’, or uncorrelated signal. Since it could be argued that the system’s primary role is to make sense of an input—whatever it may be—such overall stimulation can increase the likelihood of false correlations over space (and time); the perceptual consequence of this may be to see more than is present in the input.

Consideration of the statistical properties of natural scenes has been fruitful for understanding the way the visual system works [62–65]. The observation that natural scenes possess an approximately 1/*f* amplitude spectra and that the visual system may be particularly well adapted to this is used here to develop more ‘natural’ stimuli, to give greater ecological validity to the task [61, 66, 67]. To distinguish the task we refer to it as the Perception of Meaning task (or *POM* where the use of an acronym does not compromise readability).

Consistent with the literature reviewed above, we suggest that positive-psychotic personality traits (as measured by the Unusual Experiences sub-scale of the Schizotypy metric) mediate the occurrence of visual false alarms, and that using ecologically valid stimuli in an appropriately controlled psychophysical paradigm will enable this to be measured more effectively in a normal population. We expect that those individuals which score highly on Unusual Experiences will experience more visual false alarms and that our paradigm will allow us to discriminate between sensitivity and bias in the subjects, as well as removing any influence of suggestibility on the data.

## General Methods

Two experiments will be reported here, both examine the interaction between personality and perception. The first will pilot the *Perception Of Meaning* task and critically compare it to its closest predecessor, Jakes and Hemsley’s (1986) *Random Dots Task* outlined in the introduction. The second experiment will examine performance on a modified and improved signal-detection style version of the *POM*, and concurrently investigate the role of participants’ expectations of the stimuli on perception [12, 13, 16]. Common aspects of the methodology with be described in this section, with the complementary specifics at the head of each Experiment.

### Participants

Experiment 1 comprised 102 undergraduate psychology students (68% females) who took part in exchange for course credit. Ages ranged from 16 to 44 years (*M* = 20.0 years, *SD* = 4.6). Experiment 2 comprised 95 undergraduate psychology students (72% females) ranging in age from 17 to 41 years (*M* = 19.8 years, *SD* = 4.3). None of the participants in Experiment 2 had taken part in Experiment 1. Both studies were approved by the Human Ethics Advisory Group at the University of Melbourne and each participant provided written consent for participation and publication of their (anonymous) data.

### Questionnaire measures

Participants in Experiment 1 completed the *Oxford-Liverpool Inventory of Feelings and Experiences (O-LIFE)* [68], which is a 108-item *yes*/ *no* measure of *schizotypy* with four subscales (*Unusual Experiences ‘UnEx’* for positive-psychotic, *Cognitive Disorganization ‘CogDis’* for positive-disorganized; *Introvertive Anhedonia ‘IntAnh’* for negative; and *Impulsive Nonconformity ‘ImpNon’* for the impulsive/ antisocial subtype of *schizotypy*). The *Vividness of Visual Imagery Questionnaire (VVIQ)* [69], and *Gudjonsson’s Scale of Interrogative Suggestibility* (*GSIS*) [70] were also administered as control measures. The *VVIQ* is a 16-item questionnaire that asks respondents to mentally conjure and rate the vividness of features in four visual scenes (the face of a friend, the rising sun, a familiar shop-front, and a country scene). During this portion of the procedure, subjects were free to close their eyes, or keep them open; most subjects closed their eyes (see note in Experiment 1, discussion). The *GSIS* is an interview-style measure where respondents are read a short vignette and are then asked 20 questions relating to the story, 15 of which are leading questions regarding information that was never provided. Regardless of their accuracy, respondents are firmly told they have made a number of errors and asked the same questions again, with instructions to try and be more accurate. Responses are scored on the basis of accurate *Recall* (20 items) and two aspects of suggestibility: *Yield* (15 items)–the number of leading questions initially responded to in the leading direction, and *Shift* (15 items)–the number of leading questions the respondent changed their answer to upon being asked the second time. A total *GSIS* suggestibility score can be calculated by summing the *Yield* and *Shift* scores.

In Experiment 2, participants completed only the *UnEx*, *CogDis*, and *ImpNon* subscales of the *O-LIFE*. *IntAnh* was omitted due to time constraints and because it was of least theoretical interest.

### Psychophysical tasks and equipment

All image manipulation, coding, and presentation of experiments was carried out using the *Matlab* computer language [71] and the Psychophysics Toolbox [72]. All images were 8-bit monochrome greyscale, containing up to 256 shades of grey, ranging from black to white. The images were composed of a square matrix of pixels (1024 × 1024 (16 deg square) for Experiment 1, and 512 × 512 (8 deg square) for Experiment 2). The effective pixel size (spatial resolution) was the same for each experiment and a property of the generation hardware, but the overall image size was restricted by the database used in each case (Experiment 1 [73], Experiment 2 [74]). Stimuli were presented on a 23-inch (1920 × 1200 pixel resolution at 60 Hz, with a mean luminance of 40 cd/m^2^) *Apple* Cinema Display monitor, powered by a G4 *Apple* PowerBook. The voltage to luminance relationship of the display was approximately linear over the range used through the Apple standard gamma correction; this correction was considered adequate given the nature of the stimuli and task of the observer. Later work presenting the images on a fully calibrated CRT display has yielded similar data [43, 75, 76]. Presentation took place in a darkened room with participants seated at a distance of 964 mm from the screen supported with a chin-rest, such that one degree of visual angle corresponded to 64 screen pixels. Experimental sessions were conducted one-to-one and lasted approximately 90 minutes.

### Image generation—general

Monochrome pictures were combined with artificially generated two-dimensional noise to create visually degraded images. Experiment 1 combined the image and noise through the standard practice of addition of a given proportion of noise to an ‘image’ value within each pixel [23, 77]. Experiment 2, however, in an important variation from Experiment 1, spatially degraded the image on an alternate pixel by pixel basis. This novel spatial degradation process meant that pre-specified proportions of image pixels were randomly designated as either signal or noise. Thus, what was degraded was the degree to which the individual signal pixels correlate across space to generate a meaningful representation (Experiment 2), rather than the degree to which a single pixel in space is able to represent the signal (Experiment 1). The extent to which the visual system can discriminate between individual pixels and the precise signal to noise pixel structure will influence the effective difference between these two processes. This second method of image degradation has similarities to the phase-alignment method used recently by Hansen and colleagues [78]

**Fig 1:**
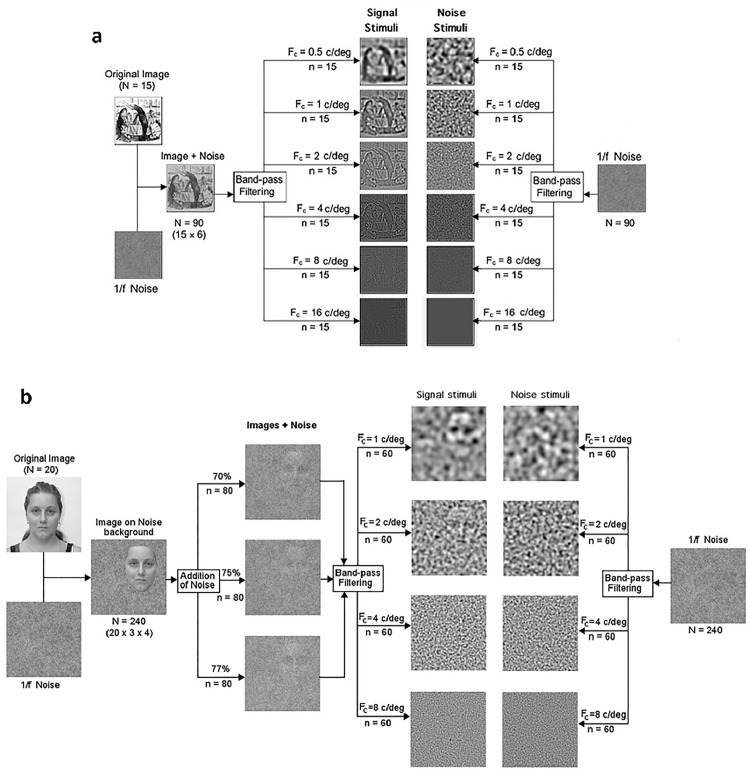
Creation of stimuli for Perception of Meaning (POM) task. Flow diagram representing the creation of the stimuli for the POM task used in Experiment 1 (1a) and Experiment 2 (1b). F_C_ = central frequency.

To create each stimulus pair, a normalised 8-bit monochrome (meaningful) signal image was randomly positioned within a pixel grid of random 2D noise of similar dimensions and mean luminance and with an amplitude spectra of 1/*f* (where *f* is the spatial frequency) to create pink-noise (as opposed to a a flat amplitude spectrum across spatial frequency, termed white-noise). A companion noise-only image was also created with the same mean and range of pixel values and the same RMS contrast. These were then combined to create a composite image where the noise was added to the signal pixel values and then normalised (Experiment 1), or a percentage of pixels were from the signal image and the remainder from the noise image (Experiment 2), again with the same RMS contrast to remove contrast as cue to the presence of an image. A further noise-only image was also paired with each signal image as its non-signal counterpart. The image pairs were then band-pass filtered (using a Fast Fourier Transform and Gaussian filter in *Matlab*) in the spatial frequency domain to contain different octave bands of spatial frequencies, centred at (0.5), 1, 2, 4, 8, or (16) c/deg at the specified viewing distance (frequencies in parentheses used in Experiment 1 only). This process is summarised in Fig 1a and 1b and experiment-specific details given below.

### Approach to the data and analysis

In this work we are interested in how different aspects of personality affect performance on the psychophysical tasks. As outlined in the introduction we have the most theoretical interest in the positive dimension (*UnEx* and *CogDis* in the O-LIFE), although we did examine all dimensions at the outset, and where possible we examined and included all dimensions of the O-LIFE, despite being aware of differing views as to the relevance of all four [40, 41]. We also analyse and present the data in a way consistent with our theoretical approach to the study; that personality is related to psychophysical performance in our particular task.

Furthermore, we analyse the data both from a continuous and dichotomous perspective to facilitate comparison with previous work [16, 45]. In order to designate respondents as low or high on a given schizotypal dimension a median-split was computed on the scores in order to compare our results to previous work. We are mindful of the potential issues of conducting a median split on continuous data e.g. [79–82], and perform regression analyses to ensure we do not rely on a single method of data treatment, while maintaining some consistency with previous work. We examined the data in all regression analyses for multi-collinearity and in no instance was the variance inflation factor (VIF) greater than 2 or the tolerance less than 0.6, indicating that multi-collinearity was not an issue. We have chosen to report both the continuous and dichotomous results in most cases. Missing data was accounted for on an analysis-by-analysis basis, which accounts for minor variations in subject number (n) in some analyses. Finally, we provide the Bayesian statistical analysis of the critical data as Supporting Information (S1 Appendix).

## Experiment 1

### Methodological specifics

#### Procedure

The two visual stimulus sets used in Experiment 1 were an adaptation of the *Random Dots Task or RDT* [45], and the newly developed *Perception Of Meaning* (*POM*) task. Similar instructions were given for completing both tasks, with participants told that they would be seeing a number of images, some of which contained something meaningful, and to describe out loud what they saw if and when they saw something meaningful or recognisable. Each image appeared on screen for 6 seconds before automatically changing to the next, for a total presentation time of approximately 18 minutes (180 stimuli in total). The order of images was randomised so each subject saw them in a different sequence. The experimenter sat about 2 metres behind the participant and recorded their responses verbatim, while remaining as unobtrusive as possible. The order of completion of the questionnaires (*O-LIFE*, *VVIQ*, and *GSIS*) and visual tasks (*RDT* and *POM*) was counterbalanced using a Latin square design, and order had no significant effects on the outcomes of interest.

#### Image generation specifics

Eight photographs of natural scenes (e.g. forests, clouds) taken from the van Hateren image database [73] and seven cartoon style line drawings (mostly of anthropomorphised animals) taken from an online database [83] formed the basis for the signal stimuli. Paired with the noise stimuli and band-pass filtered to 6 different spatial frequency brackets, the final stimulus set therefore comprised 90 signal and 90 noise stimuli (see Fig 1a), which were randomly intermixed prior to presentation such that no two participants viewed them in the same order. They were presented in a single block of 180 images. Stimuli for the *RDT* were created by generating 60 random arrays of 400 dots filling a space the same size as the images of the *POM*. From the viewing distance used, each image subtended 16° × 16° of visual angle on the retina, and was presented for 6 seconds in a rectangular temporal envelope.

#### Signal detection theory specifics

Detailed responses to the *RDT* (e.g. scenes, faces, or figures) were classified *complex* false alarms, whereas basic responses (e.g. simple geometric shapes, letters, or numerals) were classified *simple* false alarms, according to Jakes and Hemsley’s (1986) criteria. These criteria were also used to classify false alarm responses to the *POM*. In addition, misinterpreted responses to the *POM* signal stimuli that were incorrect and greatly removed from their actual content (e.g. “a dragon fighting a dinosaur” in response to an image of leaves on the ground) were also classified as false alarms using these criteria.

There were no images embedded in the RDT, so no hits or misses *per se* could be recorded although, arguably, a null response could be classified as a hit or a miss in this case. The hit rate for the POM in Experiment 1 was calculated as the total number of hits divided by the total number of signal stimuli, whereas the false alarm rate was the total number of false alarms divided by the total number of stimuli (hits + noise) to allow for the inclusion of these misinterpretations of the signal stimuli. This methodology differs from traditional signal detection methods where false alarm rates are calculated as the total number of false alarms divided by the total number of noise stimuli only. As such, the conventional rules of signal detection theory where the hit rate is equal to 1 minus the miss rate, and the false alarm rate is equal to 1 minus the correct rejection rate do not strictly apply to this data, although the relationships are a fairly close approximation.

## Results and Interim Discussion

### Descriptive statistics

Scores on the questionnaire measures are summarised in Table 1, and are all comparable to reported norms for these psychometrics [69, 70, 84, 85]. Scores on the *IntAnh* subscale of the *O-LIFE* were positively skewed and so square root transformations were computed to correct for this, and the transformed scores were used in all parametric analyses.

**Table 1:** Summary statistics (untransformed) for the questionnaire measures used in Experiment 1.

	Mean	Median	SD	Observed range
*O-LIFE*				
*Unusual Experiences*	13.3	14.0	5.6	0–26
*Cognitive Disorganization*	13.3	14.0	5.3	0–24
*Introvertive Anhedonia*	5.0	4.0	4.2	0–18
*Impulsive Nonconformity*	11.0	11.0	4.0	1–20
*GSIS*				
*Recall*	13.5	14.0	2.8	2–20
*Yield*	5.4	5.0	3.0	1–13
*Shift*	4.7	4.0	2.6	1–12
*Total*	10.0	10.0	4.7	2–20
*Vividness of Visual Imagery Questionnaire*	41.7	45.0	14.6	1–60

Note: O-LIFE = Oxford-Liverpool Inventory of Feelings and Experiences GSIS = Gudjonsson Scale of Interrogative Suggestibility.

The mean complex false alarm rates in response to the RDT (M = 11.4%, SD = 13.7) and the POM (M = 9.0%, SD = 8.5) were comparable, t(101) = .034, p > .05, and highly correlated r = .624, p < .01. The mean hit rate for the POM was 53.3% (SD = 11.0). Square root transformations were required to correct for positive skew for the complex and simple false alarm rates on both the RDT and the POM. A qualitative analysis of complex false alarm responses to the POM showed that 36% contained human faces or facial features, 25% animals or mythical creatures, 20% humanoid figures, 15% natural objects or scenes, and 4% other. It should be noted that all images in the RDT tasks were noise only, so there were no ‘hits’ or ‘misses’ *per se*, just images in which the subjects saw something or did not.

The correlations between the personality measures and performance on the two visual tasks are presented in Table 2, which indicates that positive-psychotic schizotypy (as measured by *UnEx* scores) was the only measure to be consistently associated with performance. This was reflected in the Bayesian correlation (see S1 Appendix) with a moderately high Bayes factor of 20.9, supporting the correlation between the *UnEx* score and the complex false alarms in the Perception of Meaning task.

**Table 2:** Correlations between questionnaire measures and performance on the two visual tasks used in Experiment 1.

	*UnEx*	*CogDis*	*IntAnh*	*ImpNon*	*GSIS Y*	*GSIS S*	*VVIQ*
*N*	102	102	102	102	97	97	102
*RDT* Complex FA	.215*	-.190	-.086	-.063	-.067	.164	.086
*RDT* Simple FA	.212*	-.066	-.190	.057	.031	.248*	.066
*POM* Complex FA	.327**	-.086	-.048	.085	.028	.116	-.004
*POM* Simple FA	.216*	-.074	-.140	.030	.003	.158	.071
*POM* Hits	.301**	-.030	-.229*	.156	-.018	-.054	-.053
*POM* Misses	-.376**	.115	.176	-.090	.001	-.115	.009
*POM* CR	-.323**	.042	.070	-.085	-.048	-.184	-.081

*Note*: *RDT* = *Random Dots Task*; *POM* = *Perception Of Meaning* task; FA = False alarm; CR = Correct rejection; *O-LIFE* = *Oxford-Liverpool Inventory of Feelings and Experiences* total score (subscales: *UnEx* = U*nusual Experiences*, *CogDis* = *Cognitive Disorganization*, *IntAnh* = *Introvertive Anhedonia*, and *ImpNon* = *Impulsive Nonconformity*); *GSIS* = *Gudjonsson Scale of Interrogative Suggestibility* (*Y* = *Yield* score, *S* = *Shift* score, and *T* = *Total* score—note that all correlations for the *GSIS* are partial correlations controlling for recall); *VVIQ* = *Vividness of Visual Imagery Questionnaire*.

All images in the RDT task are ‘noise only’ images, so there were not ‘hits’ or ‘misses’ per se.

* *p* < .05,

** *p* < .01 (two-tailed).

### The effect of personality on visual false alarms

Four blockwise hierarchical linear regression analyses, predicting the complex and simple false alarm rates on the *RDT* and the *POM*, were conducted. *UnEx* scores were entered in the first step, and the remaining schizotypy measures, suggestibility scores, and visual imagery scores were entered blockwise in the second step. This established whether these measures contributed to predicting false alarm rates once the effects of positive-psychotic schizotypy had been accounted for. Table 3 (left shaded columns) shows that *UnEx* scores accounted for 5% of the variance in complex responses and 4% of the variance in simple responses on the *RDT*. For complex responses, the remaining personality variables made a significant contribution in the second step, accounting for a further 19% of the variance. In the final model higher *UnEx* and *GSIS shift* scores increased, whereas higher *CogDis* scores decreased the rate of complex responses. None of the other personality measures made a significant contribution to predicting simple response rates on the *RDT* once the effects of *UnEx* had been accounted for. *UnEx* scores also made a significant contribution to predicting false alarm rates on the *POM*, accounting for 10% of the variance in complex false alarm rates and 5% of the variance in simple false alarm rates (see Table 3, rightmost columns). The addition of the remaining personality variables did not result in a statistically significant increase to the variance already accounted for by *UnEx*, either for complex or simple false alarm rates on the *POM*. It should be noted, however, that *CogDis* scores did make a significant contribution to predicting complex false alarm rates in the second step of the regression, with *POM* false alarm rates decreasing as *CogDis* scores increased. This is an unexpected result, but may possibly be related to social anxiety and neuroticism, which is reflected in an increasing *CogDis* score [86]; if this increase in anxiety causes the subjects to moderate they complex FA responses, for fear of seeming odd. We thank one of our reviewers (O.F.) for this tentative but interesting suggestion.

**Table 3:** Summary of four hierarchical block-wise regression analyses (forced entry) for personality measures predicting complex and simple false alarm rates (rows) on the *Random Dots Task* and the *Perception Of Meaning* (columns) task in Experiment 1 (*N* = 99).

	Random Dots task			Perception Of Meaning task		
	*B*	*SE B*	β	*B*	*SE B*	β
**Complex responses (false alarms)**						
Step 1.		*R*^*2*^ = .05, *p* < .05			*R*^*2*^ = .10, *p* < .005	
Constant	1.54.	0.56		1.59	0.36	
*Unusual Experiences*	0.083	0.038	.21*	.081	0.025	.32**
Step 2.		*ΔR*^*2*^ = .19, *p* < .005			*ΔR*^*2*^ = .08, *p* = .27	
Constant	4.45	2.09		2.52	1.43	
*Unusual Experiences*	0.15	0.043	.38***	0.11	0.029	.45***
*Cognitive Disorganization*	-0.15	0.043	-.38***	-0.078	0.029	-.30**
*Introvertive Anhedonia*	0.021	0.22	.009	0.097	0.15	.066
*Impulsive Nonconformity*	-0.059	0.056	-.11	-0.014	0.038	-.041
*GSIS yield*	-0.15	0.078	-.20	-0.016	0.053	-.035
*GSIS shift*	0.23	0.088	.28**	0.087	0.060	.16
*GSIS recall*	-0.052	0.082	-.069	0.012	0.056	.025
*VVIQ*	-0.49	0.80	-.062	-0.56	0.55	-.11
**Simple responses (false alarms)**						
Step 1		*R*^*2*^ = .04, *p* < .05			*R*^*2*^ = .05, *p* < .05	
Constant	0.63	0.36		1.13	0.19	
*Unusual Experiences*	0.50	0.025	.20*	0.031	0.013	.23*
Step 2		*ΔR*^*2*^ = .13, *p* = .69			*ΔR*^*2*^ = .05, *p* = .69	
Constant	0.53	1.42		1.46	0.79	
*Unusual Experiences*	0.052	0.029	.21	0.037	0.016	.27*
*Cognitive Disorganization*	-0.052	0.029	-.20	-0.023	0.016	-.16
*Introvertive Anhedonia*	-0.24	0.15	-.17	-0.046	0.085	-.059
*Impulsive Nonconformity*	0.004	0.038	.013	-0.003	0.021	-.015
*GSIS yield*	-0.032	0.053	-.069	-0.023	0.030	-.093
*GSIS shift*	0.15	0.060	.28*	0.048	0.033	.17
*GSIS recall*	0.053	0.055	.11	0.001	0.031	.004
*VVIQ*	-0.042	0.55	-.008	-0.064	0.30	-.023

Note: GSIS = Gudjonsson Scale of Interrogative Suggestibility; VVIQ = Vividness of Visual Imagery Questionnaire.

* *p* < .05,

** *p* < .01,

*** *p* < .001.

Overall, our measure of positive-psychotic schizotypy, the *UnEx* score, had the strongest association with participants’ tendency to perceive complex meaning in the random stimuli of both the *RDT* and the new *POM*. Visual imagery ability, as measured by the *VVIQ*, was not associated with performance, so it is less likely that false alarms resulted from an effortful attempt to ‘make out’ meaningful images in the noise, and plausible that they arose spontaneously in a manner analogous to visual hallucinations, especially given that their occurrence was linked to an aspect of personality suggested to lie on a continuum with the positive symptoms of psychosis. However, we note that one potential limitation of Experiment 1 was that we did not control whether participants completed the imagery task with open or closed eyes. It is possible that those completing it with closed eyes may have obtained higher *VVIQ* scores, which may have contaminated the effects of any association between imagery and schizotypy scores, or scores on the POM.

On the *RDT*, however, suggestibility also played a role, with those participants who were more likely to alter their responses due to experimenter instruction (indexed by the *GSIS Shift* scores) also more likely to report false alarms. Suggestibility did not impact performance on the *POM*. This coupled with the absence of any misleading experimenter instructions, as well as the more sophisticated and naturalistic characteristics of the noise stimuli, indicate the *POM* to be a promising tool for measuring hallucination proneness in laboratory settings with good discriminant and construct validity.

**Fig 2:**
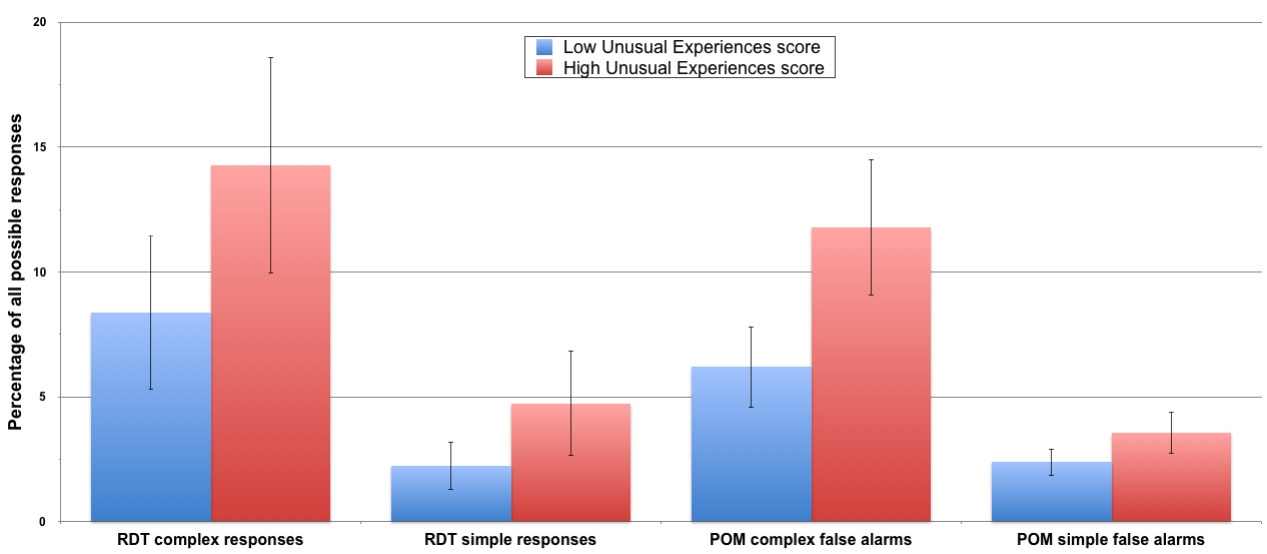
2x4 ANOVA of the rates of simple and complex false alarm responses. Response rates for simple and complex responses (false alarms) on the *Random Dots Task* (*RDT*) and the *Perception Of Meaning* task (*POM*), shown separately for individuals scoring below and above the median on the *UnEx* subscale of the *O-LIFE* (*N* = 102, error bars represent ±95% confidence intervals).

For consistency with previous work, and clarity of presentation, a 2 × 4 ANOVA was conducted using the median split of *UnEx* scores across the 4 indicators of hallucination proneness (the simple and complex false alarm rates on the *RDT* and the *POM*). The high *UnEx* group made significantly more false alarms than the low scorers. Details are presented in the Supporting Information (S1 Appendix) but the result is summarised in Fig 2. Ultimately this is the same result as the regression above, and based on the same hierarchical linear model, but presented in a more digestible format.

### The effect of spatial frequency-band on visual false alarms

**Fig 3:**
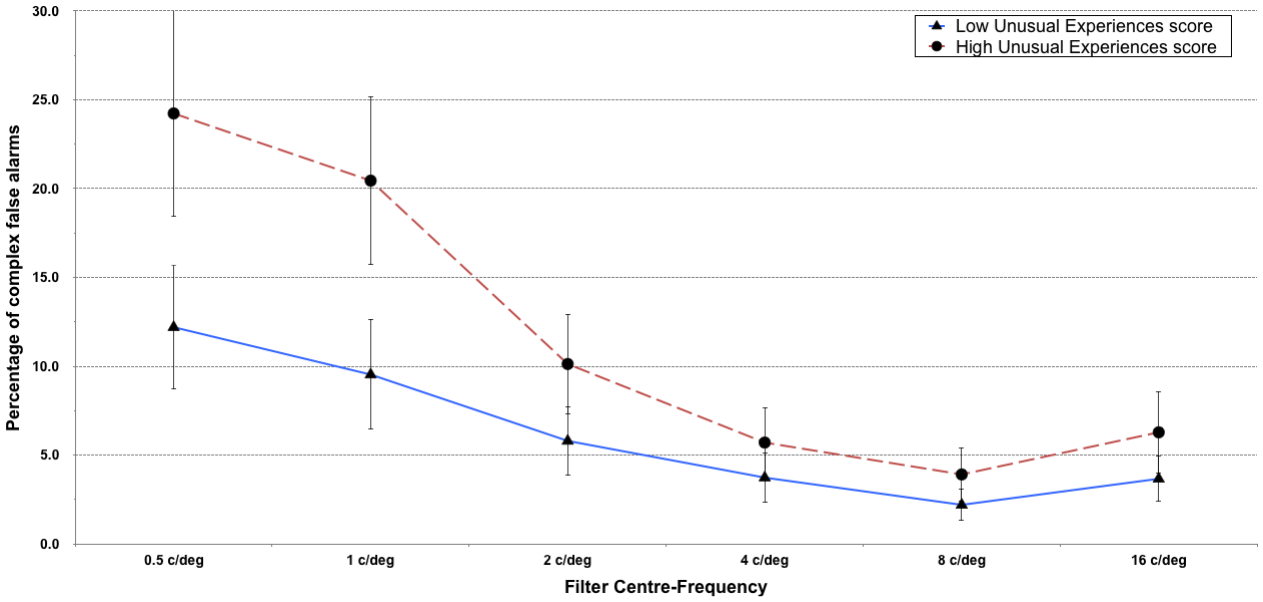
2x6 ANOVA of the rates of complex false alarm responses as a function of spatial frequency. Response rates for complex false alarms on the *Perception Of Meaning* task (*POM*) across the 6 spatial frequency bands, shown separately for individuals scoring below and above the median on the *UnEx* subscale of the *O-LIFE* (*N* = 102, error bars represent ±95% confidence intervals).

The final analysis was concerned with investigating the effect of positive-psychotic schizotypy on the complex false alarm rates on the *POM* across the different spatial frequency bands. A 2 (median split of *UnEx*) × 6 (spatial frequency brackets) ANOVA was conducted to address this question and the results are presented in Fig 3. A violation of the assumption of sphericity was indicated by Mauchly’s test, χ^*2*^(14) = 91.9, so Greenhouse-Geisser adjustments (ε = .688) were made to the degrees of freedom. A significant main effect of spatial frequency was observed, *F*(5, 500) = 70.3, *p* < .001, η_*p*_^*2*^ = .413, reflecting the fact that complex false alarms were more prevalent at the lower spatial frequencies. The interaction between *UnEx* and spatial frequency was also significant, *F*(5, 500) = 4.3, *p* < 005, η_*p*_^*2*^ = .041. Post-hoc comparisons revealed the differences between the low and high *UnEx* groups to be significant at 0.5 c/deg and 1 c/deg (*p* < .001) and also at 2 c/deg and 16 c/deg (*p* < .05). The presence of the U-shaped curve was corroborated by a statistically significant quadratic trend in complex false alarm rates with increasing spatial frequency, *F*(1, 100) = 37.2, *p* < .001, η_*p*_^*2*^ = .271.

Lower frequency bands elicited more false alarms than higher ones, an effect that was exaggerated for individuals high on *UnEx*. There is some evidence that low spatial frequencies are processed more rapidly than higher ones [87], although this will likely depend upon the precise task at hand. In addition, according to some theories (e.g. [58]), hallucinators take less time to form perceptual judgements, a similar effect having been observed in non-clinical populations where belief in the paranormal was associated with responding earlier (when less information was available) and making incorrect identifications of image-series decreasing sequentially in their level of degradation [27]. We speculate that this earlier response might therefore be exaggerated with low spatial frequency information.

Overall, the results of Experiment 1 showed that the noise-based (POM) stimuli amplified the effect originally seen for the dot (RDT) stimuli [45]; whereby a high *UnEx* O-LIFE score was correlated to the likelihood of seeing a meaningful image when there was nothing present. This validates our contention that a more ecologically valid stimulus increases individual differences in the imposition of meaning. The second experiment extends this result to examine the same effect in a signal detection framework. We also vary the spatial frequency content of the image (as in Experiment 1) and the observer’s expectation that there will be a meaningful image present [16].

## Experiment 2

### Methodological specifics

#### Procedure

All participants completed the *UnEx*, *CogDis*, and *ImpNon* subscales of the *O-LIFE* prior to the *POM*, so they could be pseudo-randomly allocated into one of three experimental conditions, ensuring an even distribution of *UnEx* scores within each group. In order to manipulate subjects’ expectations, prior to commencing the *POM*, participants were told to expect 25% (Group 1), 50% (Group 2: the control condition), or 75% (Group 3) of the stimuli in the *POM* to contain a face; in reality, 50% of stimuli contained a face for all three groups. Responses were made on the computer keyboard, where participants were instructed to press the ‘L’ key if they detected a face and the ‘A’ key if they did not (these keys were reversed for half the participants). The importance of accuracy and speed were both emphasised, and reaction times were recorded. It should be noted that responses were made on a laptop with an integrated keyboard so the losses in reaction time accuracy associated with a peripheral USB keyboard were avoided. The reaction times were also in accord with those of another study, measured using separate and calibrated button boxes (RTBox and a Cedrus box) [43, 76].

**Fig 4:**
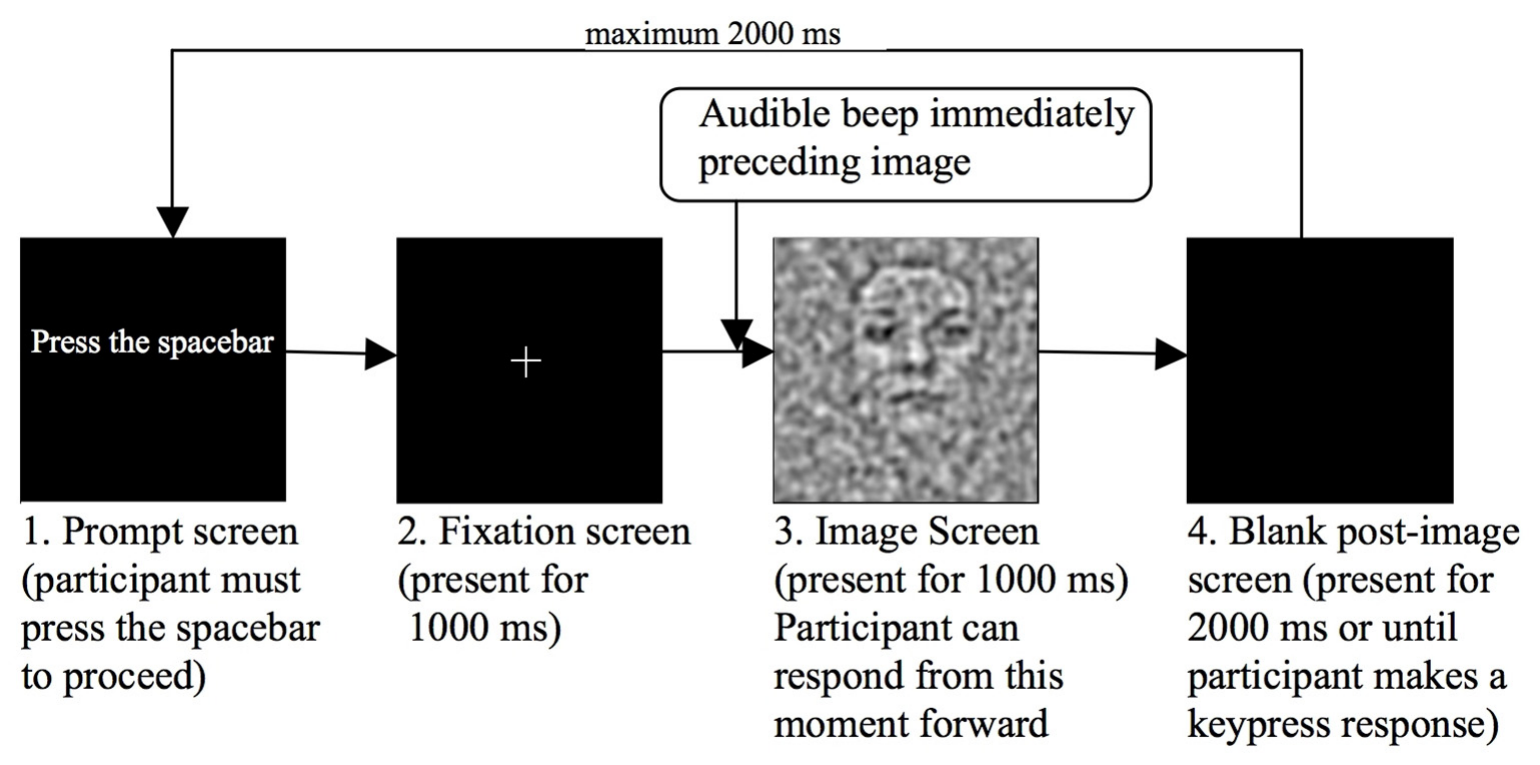
Schematic diagram of the presentation sequence for a single trial during the *Perception Of Meaning* task used in Experiment 2.

The practice block of the *POM* was administered first, during which accurate audible feedback was provided. Each stimulus trial commenced with a blank screen prompting participants to press the spacebar, a fixation cross on a black screen of mean luminance then appeared for 1000 ms. An audible beep signalled the onset of the stimulus, which was presented for exactly 1000 ms (rectangular temporal envelope), followed by another blank screen. The stimulus remained on screen for exactly 1000 ms, regardless of when the participant made their response; this is represented in Fig 4. The stimuli were presented for a shorter duration than Experiment 1, and were also only 8 deg square. Participants were able to respond at any time during the 3000 ms interval following the onset of the stimulus. Responses made after this time were not recorded. Only the practice block gave feedback as to the observers response, and the subjects were not informed how many faces to expect until after the practice block. In the test trials there was no feedback and signal stimuli were present 50% of the time in all conditions, despite the three expectation groups.

#### Image generation specifics

The *POM* task used in Experiment 2 was modified to allow for signal detection analysis so performance could be separated into factors relating to sensitivity (*Dˈ*) and response criterion (*β*) [88, 89]. This required that participants respond in a uniform manner to all signal stimuli, rather than describing what they saw, so the content of the signal stimuli had to be homogeneous. Faces were chosen for this purpose, partly because they were the most predominant spontaneous response to the open-ended *POM* used in Experiment 1. Twenty frontal photographs of human faces (10 male and 10 female) with neutral expressions, ranging in age from 19 to 37 years, taken from the database of the Productive Ageing Laboratory [74] formed the basis of the signal stimulus set. Each face was manually cropped to contain only the facial features without the hair or neck, and then pseudo-randomly positioned within a square background of 1/*f* noise, such that it was not necessarily in a central or uniform location. A typical face subtended 3.2° × 4° of visual angle within an 8° × 8° background. As described above, in a departure from the standard procedure adopted in Experiment 1, signal stimuli were degraded by pixel replacement rather than addition, with the percentage of signal pixels replaced by noise being 70, 75, or 77%. This range of stimulus image degradation was chosen to cover an approximate range close to threshold (image just visible for prolonged exposure through pilot observation). This varied for each specific image and each observer, but given the group design was a necessary step to allow presentation of a greater number of noise/image combinations which, in turn, we felt would facilitate the false-alarm response. Given the number of hits measured we were reassured this restricted range was adequate.

Images were degraded through the pixel replacement procedure prior to band-pass filtering, and only 4 spatial-frequency bands were used (centred at 1, 2, 4, or 8 c/deg) for Experiment 2. The final stimulus set thus comprised 240 signal and 240 noise stimuli, each one unique (see Fig 1b). To minimise participant fatigue, these were presented in 4 blocks, each comprising 120 stimuli with an even distribution of noise and spatial frequency bands. A practice block of a unique set of 96 stimuli (based on 8 new faces) was also created.

## Results and Interim Discussion

### Descriptive statistics

Brief demographics and schizotypy scores for the three experimental expectation groups are summarised in Table 4. No significant group differences were observed on any of these measures. Considering all three groups together, the mean false alarm rate for the signal detection version of the *POM* used in Experiment 2 was 23.1% (*SD* = 15.4%), whereas the hit rate was 62.6% (*SD* = 9.7%), both somewhat higher than the rates for the open-ended version of the *POM* used in Experiment 1. The mean sensitivity (*Dˈ*) score was 1.2 (*SD* = 0.5) and ranged between 0.4 and 2.2, indicating that all participants were able to distinguish the faces from the noise stimuli with some degree of accuracy. The mean response criterion (*β*) score was 2.0 (*SD* = 1.8) and ranged between 0.6 and 10.8. A score of *β* = 1 represents no response bias for an ideal observer. In our sample, 29.5% of participants obtained a *β* score below 1, indicating a bias toward responding as having seen a face, with the remainder scoring above 1, indicative of a bias toward responding as not having seen a face. False alarm rates and response criterion scores were both positively skewed, so square-root and inverse transformations (FA_SQ_ = √(FA); *β*_INV_ = 1 –[1 / (*β* + 1)]), were respectively applied prior to any parametric analyses.

**Table 4:** Summary statistics (untransformed) for the questionnaire measures used in Experiment 2, stratified by % of signal stimuli expected during the *Perception Of Meaning* task.

	Group 1 Expecting 25%			Group 2 Expecting 50%			Group 3 Expecting 75%			
	*M*	*Med*	*SD*	*M*	*Med*	*SD*	*M*	*Med*	*SD*	Range
*N*	31			33			31			---
Sex	74.2% female			69.7% female			71.0% female			---
Hand preference	83.9% right hand			90.9% right hand			83.9% right hand			---
Age	19.2	19.0	2.3	20.6	19.0	6.0	19.4	19.0	3.5	17–41
*O-LIFE*										
*Unusual Experiences*	10.7	11.0	6.2	11.1	11.0	5.9	10.8	10.0	5.6	1–25
*Cognitive Disorganization*	12.1	12.0	6.0	11.6	12.0	4.9	13.0	14.0	5.3	0–23
*Impulsive Nonconformity*	10.1	10.0	4.3	10.7	11.0	4.6	11.0	11.0	4.5	2–24

*Note*: *M* = mean; *Med* = median; *SD* = standard deviation; Range = observed range; *O-LIFE* = *Oxford-Liverpool Inventory of Feelings and Experiences*.

There were no significant differences by Group on any of the variables listed.

**Fig 5:**
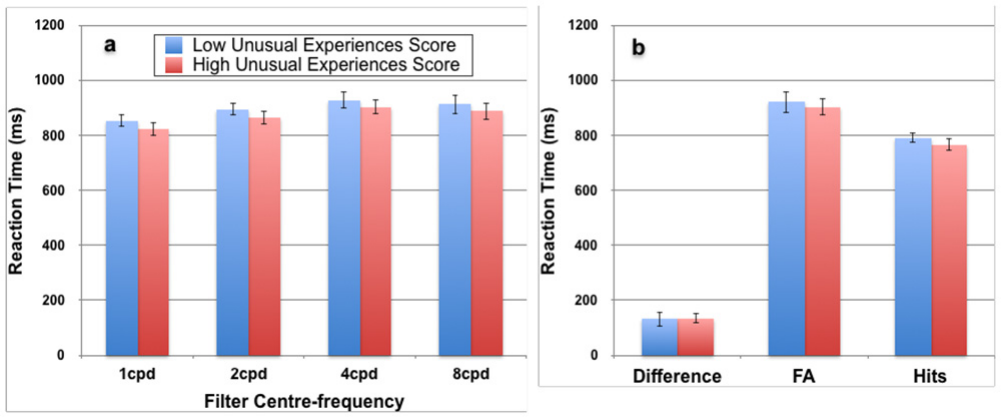
Reaction times to respond to the *Perception Of Meaning* task in Experiment 2. Results are shown separately for individuals scoring below and above the median on the *UnEx* subscale of the *O-LIFE* (*N* = 94, error bars represent 1 SEM). Panel a plots the average reactions times to respond to all trails for the two subject groups at each spatial frequency band. Panel b plots the time to respond with a False Alarm (FA) and a Hit, and the average difference between the two for each subject (Difference) collapsed across spatial frequency.

The mean reaction time (RT) was 778 ms (*SD* = 141 ms) for hits and *M* = 911 ms (*SD* = 244 ms) for false alarms. We computed a difference score for each individual by subtracting their RT for hits from their RT for false alarms, such that a positive value would indicate faster RTs for hits than false alarms. The mean difference score was 133 ms (*SD* = 166 ms), a value significantly greater than 0, *t*(94) = 7.8, *p* < .0001, indicating that a speed-accuracy trade-off was unlikely to be occurring during the *POM*, as correct responses (hits) were faster than incorrect ones. RT difference scores did not differ by *UnEx* scores, *t*(94) = 0.11, *p* > .05. We also conducted *t*-tests to compare the mean reaction times for hits and false alarms, and at the four different spatial frequencies for participants below and above the median on *UnEx*. While the high *UnEx* group did tend to respond faster, none of the differences were statistically significant (all *p* > .05). These data are plotted in Fig 5.

### The effect of personality on POM performance

**Fig 6:**
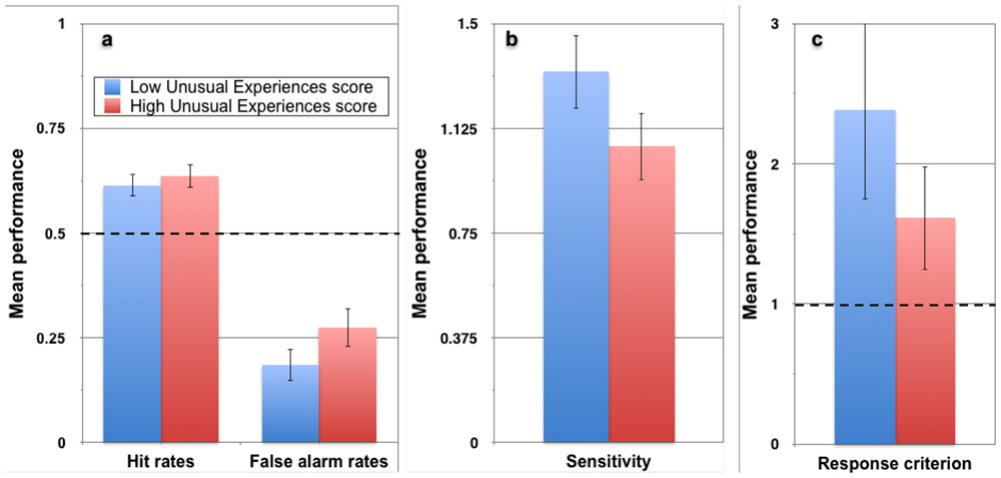
Mean performance on the *Perception Of Meaning* task in Experiment 2. Data shown separately for individuals scoring below and above the median on the *UnEx* subscale of the *O-LIFE* (*N* = 94, error bars represent ±95% confidence intervals). The reference line at 0.5 in panel a represents chance performance in the yes-no task, whereas the reference line at β = 1.0 in panel c represents the point of no response bias for an unbiased responder. A β value less than 1 indicates a bias toward saying a face was present in a noise-only stimulus.

To assess the effects of schizotypy and expectations on *POM* performance, four blockwise hierarchical linear regression analyses were conducted. Results are shown in Table 5. Only *UnEx* scores were entered in the first block, to observe the unadjusted effects of positive-psychotic schizotypy. Higher *UnEx* scores were associated with significantly more false alarms, lower sensitivity, and lower response criterion, but no difference in hit rates. These effects, collapsed across the 3 expectation conditions, are shown graphically in Fig 6, where *POM* performance is shown separately for those scoring below and above the median on *UnEx*.

**Table 5:** Summary of four hierarchical blockwise regression analyses (forced entry) for schizotypy measures and expectation predicting hit rates, false alarm rates, sensitivity (D') and response criterion (β) on the *Perception Of Meaning* task in Experiment 2 (*N* = 95).

	Hit rate			False alarm rate		
	*B*	*SE B*	β	*B*	*SE B*	β
Step 1.		*R*^*2*^ = .02, *p* = .13			*R*^*2*^ = .09, *p* < .005	
Constant	0.60	0.02		0.36	0.04	
*Unusual Experiences*	0.003	0.002	.15	0.009	0.003	.30**
Step 2.		*ΔR*^*2*^ = .0002, *p* = .99			*ΔR*^*2*^ = .02, *p* = .33	
Constant	0.60	.03		0.33	0.05	
*Unusual Experiences*	0.003	0.002	.16	0.007	0.004	.24*
*Cognitive Disorganization*	0.0001	0.002	.004	0.006	0.004	.18
*Impulsive Nonconformity*	-0.0004	0.003	-.02	-0.002	0.005	-.06
Step 3.		*ΔR*^*2*^ = .17, *p* < .0005			*ΔR*^*2*^ = .26, *p* < .0001	
Constant	0.58	0.03		0.32	0.05	
*Unusual Experiences*	0.003	0.002	.20	0.008	0.003	.28**
*Cognitive Disorganization*	-0.001	0.002	-.04	0.004	0.003	.14
*Impulsive Nonconformity*	-0.001	0.003	-.04	-0.004	0.004	-.10
Expectation 25% versus 50%	-0.004	.02	-.02	-0.07	0.03	-.19
Expectation 75% versus 50%	0.08	0.02	.40***	0.14	0.03	.40***
		**Sensitivity (D')**			**Response criterion (**β**)**	
Step 1.		*R*^*2*^ = .08, *p* < .005			*R*^*2*^ = .08, *p* < .005	
Constant	1.43	0.10		0.67	0.03	
*Unusual Experiences*	-0.02	0.01	-.29**	-0.01	0.002	-.29**
Step 2.		*ΔR*^*2*^ = .04, *p* = .17			*ΔR*^*2*^ = .02, *p* = .42	
Constant	1.54	0.13		0.69	0.04	
*Unusual Experiences*	-0.02	0.01	-.21	-0.005	0.002	-.24
*Cognitive Disorganization*	-0.02	0.01	-.22	-0.004	0.003	-.16
*Impulsive Nonconformity*	0.01	0.01	.06	0.001	0.004	.05
Step 3.		*ΔR*^*2*^ = .18, *p* < .0001			*ΔR*^*2*^ = .22, *p* < .0001	
Constant	1.51	0.13		0.69	0.04	
*Unusual Experiences*	-0.02	0.01	-.24*	-0.01	0.002	-.27*
*Cognitive Disorganization*	-0.02	0.01	-.20	-0.003	0.003	-.12
*Impulsive Nonconformity*	0.01	0.01	.10	0.003	0.003	.09
Expectation 25% versus 50%	0.21	0.10	.22*	0.06	0.03	.20*
Expectation 75% versus 50%	-0.26	0.10	-.27**	-0.09	0.03	-.34**

* *p* < .05,

** *p* < .01,

*** *p* < .001.

In the second block of the regression, entering the remaining two schizotypy measures (*CogDis* and *ImpNon* in this experiment) into the regression equation did not make any significant contribution to the predictive power of the unadjusted model when only *UnEx* was included (*p* for all Δ*R*^*2*^ > .05). Notably however, the addition of *CogDis* and *ImpNon* reduced the unique influence of *UnEx* on sensitivity (D’) scores to be statistically insignificant. This effect was largely due to the shared variance between *CogDis* and *UnEx*, with both variables in isolation being significantly associated with a reduction in sensitivity on the *POM* (*B* = -0.02, *SE B* = 0.008, *β* = -.30, *R*^*2*^ = .09, *p* < .005, for the unadjusted regression model for *CogDis* predicting *Dˈ (as opposed to UnEx as shown in* Table 5*)*), but neither making a significant contribution when considered together. Indeed, the correlation between *UnEx* and *CogDis* for the sample in Experiment 2 was *r* = .478, *p* < .001, indicating around 23% shared variance. However, the tolerance (*UnEx* = .659, *CogDis* = .714) and Variance Inflation Factor (*UnEx* = 1.518, *CogDis* = 1.400) diagnostics indicated that multicollinearity was not an issue and correlations of this magnitude between the *UnEx* and *CogDis* scales are consistent with published norms [85].

In the third and final block, dummy variables for the 25% and 75% expectation conditions were entered into the regression equation, with the 50% condition used as the reference category. Expectations strongly predicted all four performance measures on the *POM*, over and above participants’ schizotypy scores (*p* for all Δ*R*^*2*^ < .0005), with the higher expectation condition having relatively greater influence (see Table 5). Compared to the 50% control condition, expecting fewer signal stimuli on the *POM* significantly increased sensitivity and response criterion scores, but did not significantly impact hit or false alarm rates. In contrast, expecting more signal stimuli than were presented significantly increased hit and false alarm rates, but lowered sensitivity and response criterion scores. Importantly, the association between positive-psychotic schizotypy (*UnEx*) and performance on the *POM* remained significant even after the effect of expectations had been accounted for. In the final model, a one standard deviation increase in *UnEx* scores resulted in a .28 standard deviation increase in false alarm rates, a .24 standard deviation decrease in sensitivity scores, and a .27 standard deviation decrease in response criterion scores.

**Fig 7:**
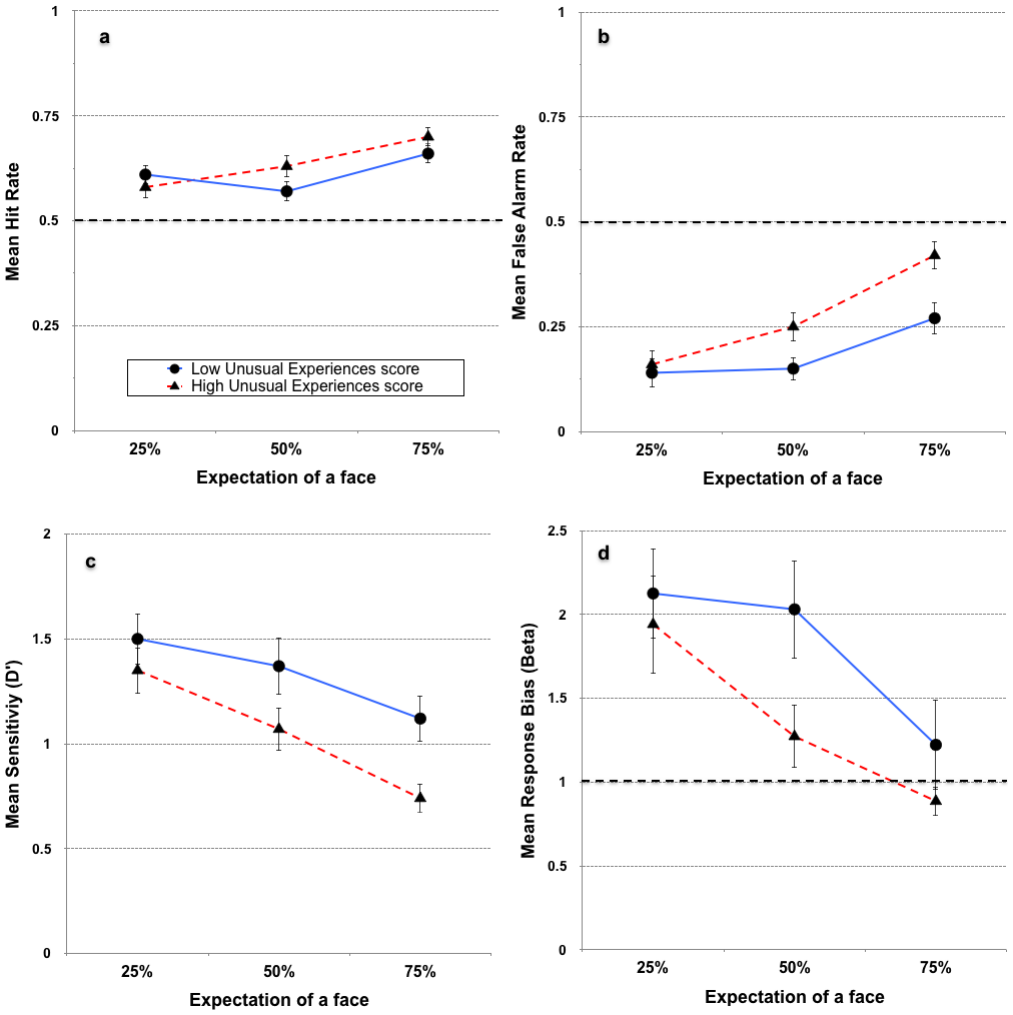
Performance across the different expectation conditions, collapsed across spatial frequency, in the *Perception Of Meaning* task. Data plotted separately for individuals scoring below and above the median on the *UnEx* subscale of the *O-LIFE* (*N* = 94, error bars represent ±95% confidence intervals). The reference lines in panels a and b represent chance performance (yes-no task), whereas the reference line at β = 1 in panel d represents the point of no response bias for an unbiased responder. A β value less than 1 indicates a bias toward saying a face was present in a noise-only stimulus.

We also explored the interaction effects between schizotypy and the three expectation conditions (2x3 mixed ANOVA), using dichotomous versions of each schizotypy variable based on median splits. We did this particularly to enable a comparison between our findings and the study of Cella et al., (2007). Unlike Cella et al. (2007), however, we did not find any significant interaction effects for any of the schizotypy variables. In fact, whereas Cella et al., (2007) found that the high *UnEx* group was especially likely to make more false alarms in the low expectation condition, our results indicated a non-significant trend in the opposite direction, with the high *UnEx* group more likely to make false alarms in the *high* expectation condition. Despite the non-significant interactions, data from the analysis are plotted in Fig 7 (collapsed across spatial frequency brackets). What the data *do* confirm are the reduced sensitivity and bias toward seeing a face (a lower *β* value) in the higher positive-psychotic (*UnEx*) group compared to the lower group.

### The effect of spatial frequency-band on POM performance

**Fig 8:**
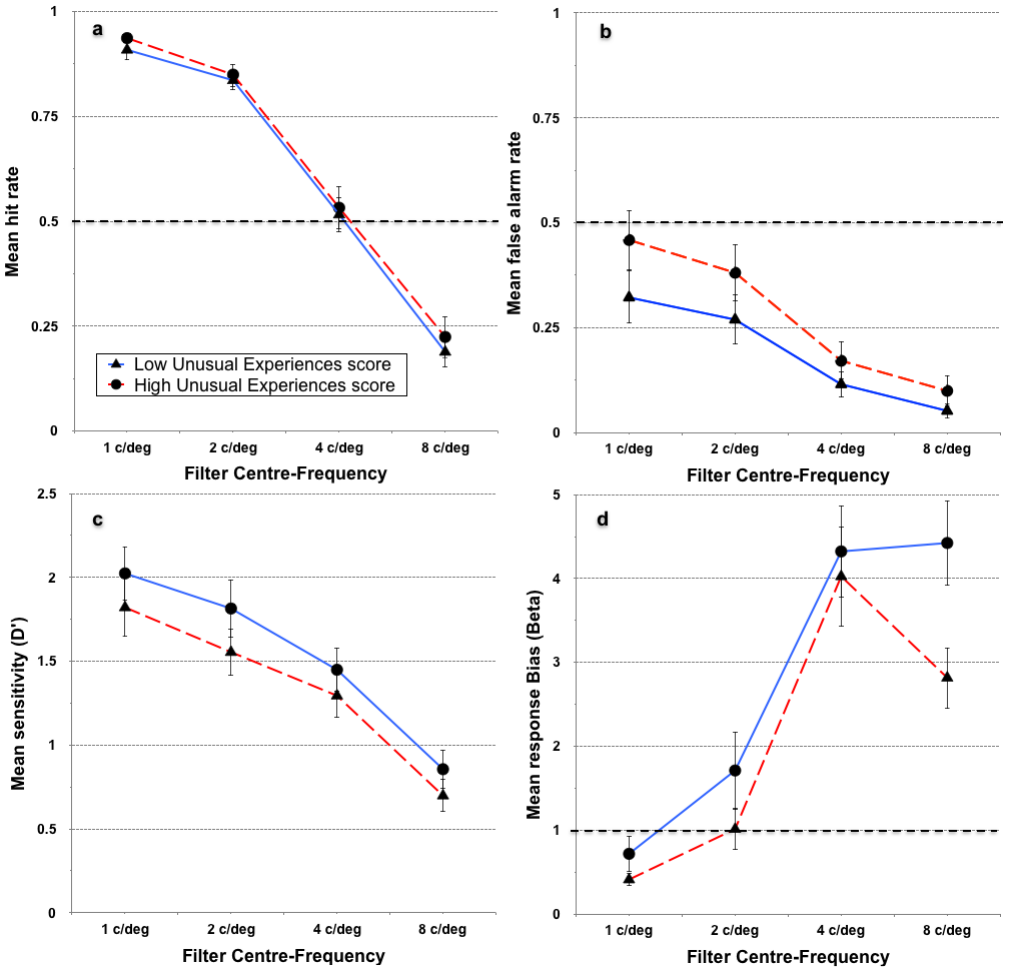
Performance across the different spatial frequency brackets, collapsed across expectation, of the *Perception Of Meaning* task. Data plotted separately for individuals scoring below and above the median on the *UnEx* subscale of the *O-LIFE* (*N* = 94, error bars represent ±95% confidence intervals). The reference lines in panels a and b represent chance performance (yes-no task), whereas the reference line at β = 1 in panel d represents the point of no response bias for an unbiased responder. A β value less than 1 indicates a bias toward saying a face was present in a noise-only stimulus.

Finally, the role of spatial frequency on *POM* performance for individuals scoring below and above the median on positive-psychotic schizotypy (collapsed across the three expectation conditions) was examined. On the basis of Experiment 1, it was anticipated that schizotypy differences would be greatest at the lower spatial frequencies. Accordingly, the interaction between *UnEx* and spatial frequency was of primary interest, though the main effect of spatial frequency on *POM* hit rates, false alarm rates, sensitivity, and response criterion will be described first. The analyses comprised 2 (*UnEx* median split) × 4 (spatial frequency brackets) mixed ANOVAs, one for each *POM* performance measure, and are presented in Fig 8. In all cases, Mauchly’s test indicated violations of the assumption of sphericity, so Greenhouse-Geisser corrections (ε = .637, .625, .887, and .804, for hit rates, false alarm rates, *Dˈ*, and *β*, respectively) were applied.

Hit rates decreased significantly with increasing spatial frequency, *F*(3, 276) = 1247.7, *p* < .001, η^*2*^_*P*_ = .93, in a significant linear trend, *F*(1, 92) = 1884.7, *p* < .001, η^*2*^_*P*_ = .95. Fig 8 indicates that hit rates for the highest spatial frequency bracket of 8 c/deg dropped below chance performance. Similarly, false alarm rates decreased, *F*(3, 276) = 223.5, *p* < .001, η^*2*^_*P*_ = .71 in a significant linear trend, *F*(1, 92) = 328.4, *p* < .001, η^*2*^_*P*_ = .78, although at no spatial frequency brackets were the mean false alarm rates greater than what would be expected by chance. Sensitivity scores also decreased significantly with increasing spatial frequency, *F*(3, 270) = 25.4, *p* < .001, η^*2*^_*P*_ = .68, in a significant linear trend, *F*(1, 90) = 366.9, *p* < .001, η^*2*^_*P*_ = .80. Lastly, response criterion scores increased with spatial frequency, *F*(3, 276) = 265.0, *p* < 001, η^*2*^_*P*_ = .74, in a statistically significant linear trend, *F*(1, 92) = 493.4, *p* < .001, η^*2*^_*P*_ = .84.

Contrary to the findings of Experiment 1, there were no significant positive-psychotic schizotypy × spatial frequency interactions on hit rates, *F*(2, 276) = .034, *p* > .05, false alarm rates, *F*(2, 276) = 1.1, *p* > .05, sensitivity, *F*(2, 270) = .046, *p* > .05, or response criterion scores, *F*(2, 276) = 0.19, *p* > .05.

To summarise, the results of Experiment 2 reflect that the increased likelihood of giving a false alarm in subjects with a high *UnEx* score (Exp 1) is related to a greater bias (*β*) toward seeing a face and a reduced sensitivity (*Dˈ*) to detecting a face when present and clarifies the initial result. Both spatial frequency and expectation influence the data but there were no significant interaction effects between schizotypy and these variables.

## General Discussion

The work described in this paper examined the effect that personality has upon an individual’s perception of meaning in noisy images.

Vision, and the brain more generally, is a noisy system. The process of deciding that a given signal is meaningful at any point in time, or at any stage in the processing hierarchy, can be considered akin to a correlation in a statistical sense. Furthermore, there are both stimulus-based and task-based factors which interact to influence the degree to which any particular neural activity may be considered to be meaningful in the overall internal representation of the input.

Our work explores how the individual (as defined by the schizotypy personality scales) influences these processes in the context of identifying meaning in a spatially degraded stimulus. The results indicate that certain aspects of personality are related to a reduced sensitivity to a stimulus and to an increased likelihood of seeing something when it is not there. This reduced sensitivity is consistent with an increased internal noise level in some individuals, making it harder to see a near threshold stimulus [90]. The increased rate of false alarms, seeing something when it’s not there, is consistent with increased internal noise if it is also the case that mistakes are made more often when there is more random activity [91], and those mistakes have a perceptual consequence. This result is congruent with a similar approach taken examining the perception of paranormal believers and skeptics when asked to identify meaning in different configure stimuli and environmental situations [28, 29, 31].

The two experiments presented here showed that strongest predictor of a complex false alarm (Experiment 1) was the *UnEx* dimension of schizotypy and that both sensitivity and bias correlate with the *UnEx* dimension only when analysed in terms of signal detection theory (Experiment 2). Put simply, this means that individuals high on this scale are less sensitive to the presence of a meaning in noise, and more inclined to see meaning when none is there.

Our experiments manipulated the spatial frequency content of the images between centre frequencies of 0.5cpd and 16cpd (2 - 8cpd in Experiment 2). When the stimuli were confined to faces embedded in noise (Experiment 2), the effect of spatial frequency was not personality-specific. In other words, making the task harder (shortening the duration) and more objective (requiring a forced-choice response) had the effect of reducing the influence of the individual on the task. Since the role of spatial frequency is probably most obvious relatively early in the visual process, and the factors affecting an individual’s personality will have greater influence on perception later in the process, this is perhaps not such a surprise.

Our manipulation of expectation of the number of images containing something meaningful was analogous to Cella et al’s (2007) [16] design using a word-detection task. Our results, however, were not entirely consistent with their findings. Both experiments found that raising expectations increased the number of false alarms irrespective of schizotypy scores, but had little effect on accuracy. In the same vein, Smith, Gosselin and Schyns (2012) found that when observers were expecting to see a face in a noisy image then they often did, even if one was never presented. Related to this result, the perceived rotation of an ambiguous stimulus depends upon expectation and, to some degree, belief about the stimulus properties [20]. We did not, however, replicate Cella et al’s (2007) interaction effects between *UnEx* scores and expectation conditions, and actually observed a non-significant trend in the opposite direction; with the high *UnEx* group more likely to make false alarms when they were presented with fewer real images than they were expecting. That is, the production of false alarms in our study was congruent with the direction of expectations, whereas Cella and colleagues found that their high *UnEx* group was particularly more likely to make false alarms when they were presented with *more* real signal stimuli than they were expecting. The major distinction between the two studies is that our signal stimuli were faces, whereas Cella et al., (2007) used common words, so one explanation for the different findings may be the different neural mechanisms involved in the perception of images and words (e.g. [92]). While our *POM* simply involves detection of the signal stimulus (a face), the detection of a real word among non-words involves the added cognitive step of recognition, given that both the signal and noise stimuli are made up of identical letter components with the difference lying only in the order of their arrangement. Arguably, our task gives rise to perceptual false alarms which more closely resemble real-world visual hallucinations than the false alarms elicited during the word-detection task of Cella et al. (2007). Our findings are also more consistent with theoretical models of how expectations contribute to the emergence of hallucinations [14].

One of the motivating factors for our use of the O-LIFE personality dimensions is its relationship to hallucination and psychosis [36], and our stimuli were developed to use controlled external noise as a tool to look at the operation of a decision-making system that is inherently noisy in its operation [93]. An effect of increased cortical ‘noise’ has been commented upon in the context of cortical oscillation in a working memory task and concurrent EEG recording both in high schizotypy individuals [94] and in schizophrenia [95]. While we do not suggest that the task at hand here is directly related to the phase-locking of cortical oscillatory activity, the observation of increased noise can be considered to have more general effects outside that directly related to oscillatory activity. The same authors also commented that increased noise may be a product of dysfunctional top-down control on the activity which prevents a clear signal being shaped and being contextually modulated in an appropriate way for the task at hand [94]. Both of these influences on the resultant percept are, we suggest, affected by the current and ongoing state of the individual as the task is carried out, and are therefore likely affected by personality.

Furthermore, it has been observed that cognitive control of intentional inhibition is reduced in schizophrenia and shows a positive correlation with the severity of auditory hallucination; more severe hallucinations appears related to reduced inhibition [96]. If the dimensional relationship to schizotypy holds then we would expect to see the decrease in D' and increased bias measured in Experiment 2 as a function of reduced control or inhibition of the neural representation of the stimulus.

As mentioned above, it has been recently shown that individuals internal templates of expected stimuli, or overall motivation regarding those stimuli, will affect whether they see something when it is not there [13, 20, 97] or which of two bistable configurations are perceived [9]; thus an internal template can be thought of as being superimposed onto an activity ‘surface’ to influence what sense may be made of that activity. This is akin to a particular belief system (acting as a template) mediating the perception of meaning in meaninglessness [28, 29, 31]. Although our stimuli were different in terms of the noise structure and, importantly, the random location of the face within the image frame [13, 20, 97], the idea of internal expectation both in terms of frequency and particular spatial structure having an influence on the decision is consistent with the current data. Furthermore, as an image becomes more ambiguous, as in the case of a bistable representation of face/vase or the necker cube, there appears to be a progressively more activity in higher brain areas, measured using multivariate pattern analysis, outside those generally associated with perception [98]. This activity outside visual cortex is also correlated to the degree of delusional belief in the particular stimulus properties which in turn affect the percept of an otherwise ambiguous rotating stimulus [20]. These results further support the suggestion that personality may well have an opportunity to alter or enhance the resultant percept, particularly when the stimulus itself does not facilitate a clear decision.

## Conclusions

The principal result of this work is that the likelihood of a given individual to see meaning in a noisy image is measurably related to their personality. The particular novelty of the current result is that we have shown that this individual difference is a not a product of suggestibility, but a genuine and consistent bias in some people toward saying a stimulus is present when there is only noise. This bias is correlated to a reduced sensitivity to the actual presence of a noisy stimulus leading to the suggestion that this mistaken perception of meaning is the product of a noisier system and the measurable consequence of a false correlation of that noise at an early stage in the system. We further suggest that this may provide the basis for the development of an hallucination, and may also explain why some abstract art can be so compelling for some, and yet leave others cold.

## Supporting Information

S1 Appendix File containing details for the 2x2 Anova illustrated in Fig 2, and Bayesian Analysis of the critical data presented in the manuscript. (DOCX)

S1 Dataset Raw data and figures in manuscript. (XLSX)

## References

[1] Cropper SJ (2014) Nature makes abstract art more captivating. The Conversation. Available: https://theconversation.com/nature-makes-abstract-visual-art-more-captivating-24723: https://theconversation.com.

[2] Campbell FW and Green DG (1965) Optical and retinal factors affecting visual resolution. Journal of Physiology 181: 576–593. 5880378 10.1113/jphysiol.1965.sp007784 PMC1357668

[3] Daitch JM and Green DG (1969) Contrast sensitivity of the human peripheral retina. Vision Research 9: 947–952. 5802399 10.1016/0042-6989(69)90100-x

[4] Cropper SJ and Wuerger SM (2005) The perception of motion in chromatic stimuli. Behavioral and Cognitive Neuroscience Reviews 4: 192–217. 16510893 10.1177/1534582305285120

[5] Meese TS, Hess RF and Williams CB (2005) Size matters, but not for everyone: Individual differences for contrast discrimination. Journal of Vision 5: 928–947. 16441194 10.1167/5.11.2

[6] Roalf D, Lowery N and Turetsky BI (2006) Behavioral and physiological findings of gender differences in global-local visual processing. Brain and Cognition 60: 32–42. 16271817 10.1016/j.bandc.2005.09.008

[7] Woods RL, Colvin CR, Vera-Diaz FA and Peli E (2010) A relationship between tolerance of blur and personality. Investigative Opthalmology & Visual Science 51: 6077–6082. 10.1167/iovs.09-5013 PMC2948623 20505192

[8] Goto SG, Ando Y, Huang C, Yee A and Lewis RS (2010) Cultural differences in the visual processing of meaning: Detecting incongruities between background and foreground objects using the N400. SCAN 5: 242–253. 10.1093/scan/nsp038 19776220 PMC2894690

[9] Balcetis E and Dunning D (2006) See what you want to see: Motivational influences on visual perception. Journal of personality and social psychology 91: 612–625. 17014288 10.1037/0022-3514.91.4.612

[10] Keilp JG, Klain HM, Brodsky B, Oquendo MA, Gorlyn M, Stanley B et al (2007) Early visual information processing deficit in depression with and without borderline personality disorder. Psychiatry Research 149: 139–145. 17097149 10.1016/j.psychres.2006.09.014 PMC3804900

[11] Kent BW, Weinstein ZA, Passarelli V, Chen Y and Siever LJ (2011) Deficient visual sensitivity in schizotypal personality disorder. Schizophrenia Research 127: 144–150. 10.1016/j.schres.2010.05.013 20541911 PMC2965789

[12] Gosselin F and Schyns PG (2003) Superstitius perceptions reveal properties of internal representations. Psychological Science 14: 505–509. 12930484 10.1111/1467-9280.03452

[13] Smith ML, Gosselin J and Schyns PG (2012) Measuring internal representations from behavioural and brain data. Curr Biol 22: 191–196. 10.1016/j.cub.2011.11.061 22264608

[14] Grossberg S (2000) How hallucinations may arise from brain mechanisms of learning, attention, and volition. Journal of the International Neuropsychological Society 6: 583–592. 10932478 10.1017/s135561770065508x

[15] Kersten D, Mamassian P and Yuille A (2004) Object perception as Bayesian inference. Annual Review of Psychology 55: 271–304. 14744217 10.1146/annurev.psych.55.090902.142005

[16] Cella M, Taylor P and Reed P (2007) Violation of expectancies produces more false positive reports in a word detection task in people scoring high in unusual experiences scale. Personality and Individual Differences 43: 59–70.

[17] Lau HC (2008) A higher-order Bayesian decision theory of consciousness. Progress in Brain Research 168: 35–48. 10.1016/S0079-6123(07)68004-2 18166384

[18] Reed P, Wakefield D, Harris J, Parry J, Cella M, and Tsakanikos E. (2008) Seeing non-existent events: Effects of environmental conditions, schizotypal symptoms, and sub-clinical characteristics. Journal of Behavior Therapy 39: 276–291. 10.1016/j.jbtep.2007.07.005 17900527

[19] Rieth CA, Lee K, Lui J, Tian J and Huber DE (2011) Faces in the mist: Illusory face and letter detection. 2: 458–476. 10.1068/i0421 PMC3485785 23145238

[20] Schmack K, Gomez-Carrillo de Castro A, Rothkirch M, Sekutowicz M, Rossler H, Haynes JD et al (2013) Delusions and the role of beliefs in perceptual inference. J Neurosci 33: 13701–13712. 10.1523/JNEUROSCI.1778-13.2013 23966692 PMC6618656

[21] Yuille AL and Bulthoff HH (1996) Bayesian decision theory and psychophysics In: Knill D. C. and Richards W., editors. Perception as Bayesian inference. Cambridge: Cambridge University Press pp. 123–162.

[22] Rensink RA (2000) Seeing, sensing, and scrutinizing. Vision Research 40: 1469–1487. 10788653 10.1016/s0042-6989(00)00003-1

[23] Wild HA and Busey TA (2004) Seeing faces in the noise: Stochastic activity in perceptual regions of the brain may influence the perception of ambiguous stimuli. Psychom Bull Rev 11: 475–481. 10.3758/bf03196598 15376798

[24] Hesselmann G, Kell CA, Eger E and Kleinschmidt A (2008) Spontaneous local variations in ongoing neural activity bias perceptual decisions. Proc Natl Acad Sci U S A 105: 10984–10989. 10.1073/pnas.0712043105 18664576 PMC2504783

[25] Hesselmann G, Kell CA and Kleinschmidt A (2008) Ongoing activity fluctuations in hMT+ bias the perception of coherent visual motion. J Neurosci 28: 14481–14485. 10.1523/JNEUROSCI.4398-08.2008 19118182 PMC6671252

[26] Hesselmann G, Sadaghiani S, Friston KJ and Kleinschmidt A (2010) Predictive coding or evidence accumulation? False inference and neuronal fluctuations. PLoS One 5: e9926 10.1371/journal.pone.0009926 20369004 PMC2848028

[27] Blackmore S and Moore R (1994) Seeing things: Visual recognition and belief in the paranormal. Eur J Parapsychol 10: 91–103.

[28] Riekki T, Lindeman M, Aleneff M, Halme A and Nuortimo A (2012) Paranormal and Religious Believers Are More Prone to Illusory Face Perception than Skeptics and Non-believers. Applied Cognitive Psychology.

[29] Riekki T, Lindeman M and Raij TT (2014) Supernatural believers attribute more intentions to random movement than skeptics: an fMRI study. Social neuroscience 9: 400–411. 10.1080/17470919.2014.906366 24720663

[30] Bressan P (2002) The Connection Between Random Sequences, Everyday Coincidences, and Belief in the Paranormal. Applied Cognitive Psychology 16: 17–34.

[31] Krummenacher P, Mohr C, Haker H and Brugger P (2010) Dopamine, paranormal belief, and the detection of meaningful stimuli. J Cogn Neurosci 22: 1670–1681. 10.1162/jocn.2009.21313 19642883

[32] Eysenck HJ (1967) The biological basis of personality. Springfield, IL.: Thomas.

[33] Eckblad M and Chapman LJ (1983) Magical ideation as an indicator of schizotypy. Journal of Consulting and Clinical Psychology 51: 215–225. 6841765 10.1037//0022-006x.51.2.215

[34] Claridge G (1997) Schizotypy: Implications for illness and health. Oxford: Oxford University Press.

[35] Barrantes-Vidal N, Chun CA, Myin-Germeys I and Kwapil TR (2013) Psychometric schizotypy predicts psychotic-like, paranoid, and negative symptoms in daily life. J Abnorm Psychol 122: 1077–1087. 10.1037/a0034793 24364610

[36] Nelson MT, Seal ML, Pantelis C and Phillips LJ (2013) Evidence of a dimensional relationhsip between shizotypy and schizophrenia: A systematic review. Neuroscience and Biobehavioural Reviews 37: 317–327. 10.1016/j.neubiorev.2013.01.004 23313650

[37] McCreery C and Claridge G (1996) A study of hallucination in normal subjects—I. Self-report data. Personality and Individual Differences 21: 739–747.

[38] Chapman LJ, Chapman JP, Kwapil TR, Eckblad M and Zinser MC (1994) Putatively psychosis-prone subjects 10 years later. Journal of Abnormal Psychology 103: 171–183. 8040487 10.1037//0021-843x.103.2.171

[39] Rossler W, Hengartner MP, Ajdacic-Gross V, Haker H and Angst J (2013) Deconstructing sub-clinical psychosis into latent-state and trait variables over a 30-year time span. Schizophr Res 150: 197–204. 10.1016/j.schres.2013.07.042 23932663

[40] Mason OJ, Claridge G and Jackson M (1995) New scales for the assessment of schizotypy. Personality and Individual Differences 18: 7–13.

[41] Lin A, Wigman JT, Nelson B, Wood SJ, Vollebergh WA, van Os J et al (2013) Follow-up factor structure of schizotypy and its clinical associations in a help-seeking sample meeting ultra-high risk for psychosis criteria at baseline. Compr Psychiatry 54: 173–180. 10.1016/j.comppsych.2012.06.011 22901838

[42] Partos T (2012) Hallucination proneness in the visual domain: A spectrum of interaction between visual processing and personality Melbourne School of Psychological Sciences. Melbourne, Australia: University of Melbourne.

[43] Cropper SJ and Partos T (2014) Artists and hallucinations: creativity and schizotypy in the detection of meaning Perception VSAC Abstracts; Belgrade, Serbia.

[44] Mohr C and Claridge G (2015) Schizotypy—do not worry, it is not all worrisome. Schizophr Bull 41 Suppl 2: S436–443. 10.1093/schbul/sbu185 25810058 PMC4373632

[45] Jakes S and Hemsley DR (1986) Individual differences in reaction to brief exposure to unpatterned visual stimulation. Personality and Individual Differences 7: 121–123.

[46] Brugger P, Regard M, Landis T, Cook N, Krebs D and Neiderberger J (1993) 'Meaningful' patterns in visual noise: Effects of lateral stimulation and the observer's belief in ESP. Psychopathology 26: 261–265. 8190845 10.1159/000284831

[47] Farias M, Claridge G and Lalljee M (2005) Personality and cognitive predictors of New Age practices and beliefs. Personality and Individual Differences 39: 979–989.

[48] Tsakanikos E and Reed P (2005) Seeing words that are not there: detection biases in schizotypy. Br J Clin Psychol 44: 295–299. 16004663 10.1348/014466505X28757

[49] Phillips WA, Chapman KLS and Berry PD (2004) Size perception is less context-sensitive in males. Perception 33: 79–86. 15035330 10.1068/p5110

[50] Dakin S, Carlin P and Hemsley D (2006) Weak suppression of visual context in chronic schizophrenia. Curr Biol 15: 822–824. 10.1016/j.cub.2005.10.015 16243017

[51] Bosten JM and Mollon JD (2008) Individual differences in simultaneous contrast. Perception 37: 105.

[52] Ffytche DH and Zeki S (1996) Brain activity related to the perception of illusory contours. NeuroImage 3: 104–108. 9345481 10.1006/nimg.1996.0012

[53] McCullough C (1965) Color adaptation of edge-detectors in the human visual system. Science 149: 1115–1116. 17737844 10.1126/science.149.3688.1115

[54] Morita T, Kochiyama T, Okada T, Yonekura Y, Matsumura M and Sadato N (2004) The neural substrates of conscious color perception demonstrated using fMRI. NeuroImage 21: 1665–1673. 15050589 10.1016/j.neuroimage.2003.12.019

[55] Behrendt R and Young C (2004) Hallucinations in schizophrenia, sensory impairment, and brain disease: A unifying model. Behavioral and Brain Sciences 24: 771–830. 10.1017/s0140525x04000184 16035402

[56] Allen P, Laroi F, McGuire PK and Aleman A (2008) The hallucinating brain: A review of structural and functional neuroimaging studies of hallucinations. Neuroscience and biobehavioral reviews 32: 175–191. 17884165 10.1016/j.neubiorev.2007.07.012

[57] Friston K (2002) Functional integration and inference in the brain. Progress in Neurobiology 68: 113–143. 12450490 10.1016/s0301-0082(02)00076-x

[58] Bentall R (1990) The illusion of reality: A review and integration of psychological research on hallucinations. Psychological Bulletin 107: 82–95. 2404293 10.1037/0033-2909.107.1.82

[59] Silverstein SM, Raulin ML, Pristach EA and Pomeranz JR (1992) Perceptual organization and schizotypy. Journal of Abnormal Psychology 101: 265–270. 1583218 10.1037//0021-843x.101.2.265

[60] Feigelson EM (1984) Spatial Structure of Clouds In: Feigelson E. M., editor editors. Radiation in a Cloudy Atmosphere. Springer Netherlands pp. 4–15.

[61] Field DJ (1987) Relations between the statistics of natural images and the response properties of cortical cells. J Opt Soc Am A 4: 2379–2394. 3430225 10.1364/josaa.4.002379

[62] Olshausen B and Field D (1997) Sparse coding with an overcomplete basis set: a strategy employed by V1?. Vision Res 37: 311–325. 10.1016/s0042-6989(97)00169-7 9425546

[63] Willmore B, Watters PA and Tolhurst DJ (2000) A comparison of natural-image-based models of simple-cell coding. Perception 29: 1017–1040. 11144817 10.1068/p2963

[64] Simoncelli EP and Olshausen BA (2001) Natural image statistics and neural representation. Annu Rev Neurosci 24: 1193–1216. 11520932 10.1146/annurev.neuro.24.1.1193

[65] Simoncelli EP (2003) Seeing patterns in the noise. Trends Cogn Sci 7: 51–53. 12584015 10.1016/s1364-6613(02)00043-8

[66] Field DJ and Brady N (1997) Visual sensitivity, blur and the sources of variability in the amplitude spectra of natural scenes. Vision Res 37: 3367–3383. 9425550 10.1016/s0042-6989(97)00181-8

[67] Bex PJ and Makous W (2002) Spatial frequency, phase, and the contrast of natural images. J Opt Soc Am A Opt Image Sci Vis 19: 1096–1106. 12049346 10.1364/josaa.19.001096

[68] Mason OJ, Claridge G and Williams L (1997) Questionnaire measurement In: Claridge G., editor editors. Schizotypy: Implications for Illness and Health. Oxford: Oxford University Press pp. 19–37.

[69] Marks DF (1973) Visual imagery differences in the recall of pictures. British Journal of Psychology 64: 17–24. 4742442 10.1111/j.2044-8295.1973.tb01322.x

[70] Gudjonsson GH (1984) A new scale of interrogative suggestibility. Personality and Individual Differences 5: 303–314.

[71] Inc. TM (2006) Matlab. Natick, MA.: The MathWorks Inc.

[72] Brainard DG (1997) The Psychophysics Toolbox. Spatial Vision 10: 433–436. 9176952

[73] van Hateren JH and van der Schaaf A (1998) Independent component filters of natural images compared with simple cells in primary visual cortex. Proceedings of the Royal Society of London Series B, Biological Sciences 265: 359–366. 9523437 10.1098/rspb.1998.0303 PMC1688904

[74] Minear M and Park DC (2004) A lifespan database of adult facial stimuli. Behavior Research Methods, Instruments and Computers 36: 630–633. 10.3758/bf03206543 15641408

[75] Cropper SJ and Partos T (2011) Individual differences in the construction of meaning from noise and their relation to the development of visual hallucinations. Perception.

[76] Cropper SJ and Partos T (2011) Which way up is an Hallucination? Australian Journal of Psychology.

[77] Solomon JA and Pelli DG (1994) The visual filter mediating letter identification. Nature 369: 395–397. 8196766 10.1038/369395a0

[78] Hansen BC, Farivar R, Thompson B and Hess RF (2008) A critical band of phase alignment for discrimination but not recognition of human faces. Vision Res 48: 2523–2536. 10.1016/j.visres.2008.08.016 18801383

[79] Iacobucci D, Posavac SS, Kardes FR, Schneider MJ and Popovich DL (2015) The median split: Robust, refined, and revived. Journal of Consumer Psychology 25: 690–704.

[80] Iacobucci D, Posavac SS, Kardes FR, Schneider MJ and Popovich DL (2015) Toward a more nuanced understanding of the statistical properties of a median split. Journal of Consumer Psychology 25: 652–665.

[81] McClelland GH, Lynch JJG, Irwin JR, Spiller SA and Fitzsimons GJ (2015) Median splits, Type II errors, and false—positive consumer psychology: Don't fight the power. Journal of Consumer Psychology 25: 679–689.

[82] Rucker DD, McShane BB and Preacher KJ (2015) A researcher's guide to regression, discretization, and median splits of continuous variables. Journal of Consumer Psychology 25: 666–678.

[83] (1990–1998) Black and White Prints. IMSI MasterClips(R) and MasterPhotos(TM) Premium Image Collection.

[84] Sheehan PW (1967) A shortened form of Betts' Questionnaire upon Mental Imagery. Journal of Clinical Psychology 23: 386–389. 6082130 10.1002/1097-4679(196707)23:3<386::aid-jclp2270230328>3.0.co;2-s

[85] Mason O and Claridge G (2006) The Oxford-Liverpool Inventory of Feelings and Experiences (O-LIFE): further description and extended norms. Schizophr Res 82: 203–211. 16417985 10.1016/j.schres.2005.12.845

[86] Claridge G, McCreery C, Mason O, Bentall R, Boyle G, Slade P et al (1996) The factor structure of 'schizotypal' traits: A large replication study. British Journal of Clinical Psychology 35: 103–115. 8673026 10.1111/j.2044-8260.1996.tb01166.x

[87] Loftus GR and Harley EM (2004) How different spatial-frequency components contribute to visual information acquisition. J Exp Psych: Human Percept & Perf 30: 104–118. 10.1037/0096-1523.30.1.104 14769071

[88] Tanner WP Jr. and Swets JA (1954) The human use of information I. Signal detection for the case of a signal known exactly. Transactions of the IRE PGIT-4: 213–221.

[89] Macmillan NA and Creelman CD (1991) Detection theory: A users guide. New York: Cambridge University Press.

[90] Pelli DG and Farell B (1999) Why use noise? J Opt Soc Am A 16: 647–653. 10.1364/josaa.16.000647 10069051

[91] Green DM and Swets JA (1966) Signal detection theory and psychophysics. New York: Wiley.

[92] Rossion B, Joyce CA, Cottrell GW and Tarr MJ (2003) Early lateralization and orientation tuning for face, word, and object processing in the visual cortex. Neuroimage 20: 1609–1624. 14642472 10.1016/j.neuroimage.2003.07.010

[93] Pelli DG (1981) Effects of visual noise Experimental psychology. Cambridge: University of Cambridge, England.

[94] Koychev I, Deakin JF, Haenschel C and El-Deredy W (2011) Abnormal neural oscillations in schizotypy during a visual working memory task: support for a deficient top-down network? Neuropsychologia 49: 2866–2873. 10.1016/j.neuropsychologia.2011.06.012 21703284

[95] Winterer G, Coppola R, Goldberg TE, Egan MF, Jones DW, Sanchez CE et al (2004) Prefrontal broadband noise, working memory, and genetic risk for schizophrenia. Am J Psychiatry 161: 490–500. 14992975 10.1176/appi.ajp.161.3.490

[96] Badcock JC, Waters FA, Maybery MT and Michie PT (2005) Auditory hallucinations: failure to inhibit irrelevant memories. Cogn Neuropsychiatry 10: 125–136. 16571456 10.1080/13546800344000363

[97] Righart R, Andersson F, Schwartz S, Mayer E and Vuilleumier P (2010) Top-down activation of fusiform cortex without seeing faces in prosopagnosia. Cereb Cortex 20: 1878–1890. 10.1093/cercor/bhp254 19939884

[98] Wang M, Arteaga D and He BJ (2013) Brain mechanisms for simple perception and bistable perception. Proc Natl Acad Sci U S A 110: E3350–3359. 10.1073/pnas.1221945110 23942129 PMC3761598

