# Supplementary material for: You Don’t See What I See: Individual Differences in the Perception of Meaning from Visual Stimuli
Source: PLoS One. 2016 Mar 8;11(3):e0150615. doi: 10.1371/journal.pone.0150615 (PMC4783041; doi:10.1371/journal.pone.0150615)
Supplement: S1 Appendix — (DOCX) [file pone.0150615.s001.docx]

**Supplemental Analysis**

**Experiment 1**

**2x2 ANOVA details**

For optimum comparability with the previous work cited in the introduction, a 2 × 4 ANOVA was conducted using the median split of *UnEx* scores across the 4 indicators of hallucination proneness (the simple and complex false alarm rates on the *RDT* and the *POM*). The results are presented in Figure 2 in the main manuscript. Mauchly’s test indicated a violation of the assumption of sphericity, χ*^2^*(5) = 49.7, *p* < .001, so Greenhouse-Geisser corrections (ε = .770) were applied to the degrees of freedom. The effect of response category was significant, *F*(3, 300) = 39.3, *p* < .001, η*_p_^2^* = .282, reflecting the fact that participants made more complex than simple false alarms, in both tasks. The high *UnEx* group also made significantly more false alarms than the low *UnEx* group, *F*(1, 100) = 10.9, *p* < .005, η*_p_^2^* = .099. Post-hoc comparisons revealed that this effect was strongest for complex false alarm responses made to the *POM*, *t*(100) = 3.6, *p* < .001 (*r^2^* = .11), however the effect was significant for all four response categories at *p* < .05. If applying a Bonferroni correction for multiple comparisons (α = .0125), the effect of *UnEx* would only be significant for the complex false alarm responses to the *POM*, however, given the questionable utility and suitability of applying such corrections they will not be used for the remaining analyses [1]. The interaction effect between response category and *UnEx* scores was not statistically significant, *F*(3, 300) = 1.7, *p* = .18.

Bayesian Analysis of the principal data

We also re-analysed all critical data with Bayesian statistics in JASP [2], the results of which are presented below. The results of these analyses support our original conclusions, and strengthen the case for the principal relationship shown between the *UnEx* scale in the O-LIFE and our variables of interest, particularly the False Alarm.

The Bayesian approach allows a more nuanced and balanced view of the likelihood that a given alternative hypothesis may be true, rather than simply that the Null Hypothesis is not supported, as is the case in orthodox (‘frequentist’) statistics. This approach is becoming a more commonplace in the literature, particularly in the cognitive sciences, though by no means is it universally accepted [3-5]. The Bayes factor, the ‘score’ provided for a given analysis in place of the p-value in orthodox statistics, is defined as evidence in the data supporting one hypothesis over another. Here, the notation BF_10_ indicates the Bayes factor to quantify evidence for the alternative hypothesis (1) relative to the null hypothesis (0). The higher the Bayes factor, the stronger the support for the alternative hypothesis. In our case, a higher Bayes factor indicates a measurable relationship between the psychophysical dependent variable (e.g. False Alarms or D-Prime) and the personality subscale (e.g. *UnEx*). As a guide, the Bayes factor given for each analysis may be interpreted as follows [6]:

| **BF_10_** | **Strength of evidence** |
| --- | --- |
| 1 to 3 | not worth more than a bare mention |
| 3 to 20 | positive |
| 20 to 150 | strong |
| >150 | very strong |

| **Experiment 1: Bayesian Pearson Correlations** | | | | | | | | | | | | | | | | | | | | | | | | | | | | | |
| --- | --- | --- | --- | --- | --- | --- | --- | --- | --- | --- | --- | --- | --- | --- | --- | --- | --- | --- | --- | --- | --- | --- | --- | --- | --- | --- | --- | --- | --- |
|  | |  | | **UnEx** | | **CogDis** | | **ImpNon** | | **IntAnh** | | **VVIQ** | | **GSIS** | | **DOTs** | | **DOTc** | | **POMs** | | **POMc** | | **POM_HIT** | | **POM_MISS** | | **POM_CR** | |
| UnEx |  | Pearson's r |  | — |  | 0.342 | ** | 0.353 | ** | -0.126 |  | 0.075 |  | -0.001 |  | **0.190** |  | **0.225** |  | **0.229** |  | **0.315** | ***** | **0.165** |  | **-0.379** | ******* | **-0.323** | * |
|  |  | BF₁₀ |  | — |  | 55.35 |  | 84.276 |  | 0.271 |  | 0.163 |  | 0.124 |  | **0.747** |  | **1.581** |  | **1.724** |  | **20.861** |  | **0.478** |  | **245.901** |  | **27.750** |  |
| CogDis |  | Pearson's r |  |  |  | — |  | 0.253 |  | 0.228 |  | -0.146 |  | 0.096 |  | -0.128 |  | -0.133 |  | -0.064 |  | -0.089 |  | -0.100 |  | 0.086 |  | 0.042 |  |
|  |  | BF₁₀ |  |  |  | — |  | 3.140 |  | 1.686 |  | 0.355 |  | 0.196 |  | 0.279 |  | 0.296 |  | 0.151 |  | 0.183 |  | 0.203 |  | 0.177 |  | 0.135 |  |
| ImpNon |  | Pearson's r |  |  |  |  |  | — |  | 0.058 |  | -0.255 |  | -0.002 |  | 0.061 |  | -0.052 |  | 0.052 |  | 0.066 |  | 0.067 |  | -0.094 |  | -0.085 |  |
|  |  | BF₁₀ |  |  |  |  |  | — |  | 0.146 |  | 3.272 |  | 0.124 |  | 0.149 |  | 0.142 |  | 0.141 |  | 0.153 |  | 0.154 |  | 0.192 |  | 0.177 |  |
| IntAnh |  | Pearson's r |  |  |  |  |  |  |  | — |  | -0.062 |  | 0.096 |  | -0.171 |  | 0.030 |  | -0.155 |  | 0.001 |  | -0.204 |  | 0.183 |  | 0.073 |  |
|  |  | BF₁₀ |  |  |  |  |  |  |  | — |  | 0.150 |  | 0.196 |  | 0.534 |  | 0.129 |  | 0.405 |  | 0.124 |  | 1.002 |  | 0.659 |  | 0.160 |  |
| VVIQ |  | Pearson's r |  |  |  |  |  |  |  |  |  | — |  | -0.160 |  | 0.074 |  | 0.012 |  | 0.045 |  | 0.024 |  | -0.020 |  | 0.011 |  | -0.048 |  |
|  |  | BF₁₀ |  |  |  |  |  |  |  |  |  | — |  | 0.436 |  | 0.163 |  | 0.125 |  | 0.137 |  | 0.128 |  | 0.127 |  | 0.125 |  | 0.139 |  |
| GSIS |  | Pearson's r |  |  |  |  |  |  |  |  |  |  |  | — |  | 0.074 |  | 0.088 |  | 0.140 |  | 0.117 |  | -0.056 |  | -0.027 |  | -0.190 |  |
|  |  | BF₁₀ |  |  |  |  |  |  |  |  |  |  |  | — |  | 0.162 |  | 0.182 |  | 0.324 |  | 0.243 |  | 0.145 |  | 0.129 |  | 0.748 |  |
| DOTsimple |  | Pearson's r |  |  |  |  |  |  |  |  |  |  |  |  |  | — |  | 0.280 |  | 0.410 | *** | 0.265 |  | 0.162 |  | -0.379 | *** | -0.262 |  |
|  |  | BF₁₀ |  |  |  |  |  |  |  |  |  |  |  |  |  | — |  | 6.766 |  | 1044.563 |  | 4.344 |  | 0.458 |  | 250.062 |  | 4.092 |  |
| DOTcomp |  | Pearson's r |  |  |  |  |  |  |  |  |  |  |  |  |  |  |  | — |  | 0.291 |  | 0.631 | *** | 0.137 |  | -0.495 | *** | -0.520 | *** |
|  |  | BF₁₀ |  |  |  |  |  |  |  |  |  |  |  |  |  |  |  | — |  | 9.399 |  | 8.036e +9 |  | 0.314 |  | 117122.668 |  | 656976.743 |  |
| POMsimple |  | Pearson's r |  |  |  |  |  |  |  |  |  |  |  |  |  |  |  |  |  | — |  | 0.518 | *** | 0.098 |  | -0.458 | *** | -0.591 | *** |
|  |  | BF₁₀ |  |  |  |  |  |  |  |  |  |  |  |  |  |  |  |  |  | — |  | 576319.678 |  | 0.198 |  | 12939.856 |  | 1.707e  +8 |  |
| POMcomp |  | Pearson's r |  |  |  |  |  |  |  |  |  |  |  |  |  |  |  |  |  |  |  | — |  | 0.071 |  | -0.674 | *** | -0.898 | *** |
|  |  | BF₁₀ |  |  |  |  |  |  |  |  |  |  |  |  |  |  |  |  |  |  |  | — |  | 0.159 |  | 4.100e +17 |  | 1.751e +100 |  |
| POM_HIT |  | Pearson's r |  |  |  |  |  |  |  |  |  |  |  |  |  |  |  |  |  |  |  |  |  | — |  | -0.572 | *** | -0.111 |  |
|  |  | BF₁₀ |  |  |  |  |  |  |  |  |  |  |  |  |  |  |  |  |  |  |  |  |  | — |  | 3.356e  +7 |  | 0.228 |  |
| POM_MISS |  | Pearson's r |  |  |  |  |  |  |  |  |  |  |  |  |  |  |  |  |  |  |  |  |  |  |  | — |  | 0.571 | *** |
|  |  | BF₁₀ |  |  |  |  |  |  |  |  |  |  |  |  |  |  |  |  |  |  |  |  |  |  |  | — |  | 3.014e  +7 |  |
| POM_CR |  | Pearson's r |  |  |  |  |  |  |  |  |  |  |  |  |  |  |  |  |  |  |  |  |  |  |  |  |  | — |  |
|  |  | BF₁₀ |  |  |  |  |  |  |  |  |  |  |  |  |  |  |  |  |  |  |  |  |  |  |  |  |  | — |  |
|  | | | | | | | | | | | | | | | | | | | | | | | | | | | | | |
| * BF₁₀ > 10, ** , BF₁₀ > 30, *** BF₁₀ > 100 | | | | | | | | | | | | | | | | | | | | | | | | | | | | | |

**Experiment 1: Bayesian Correlation plot**

**
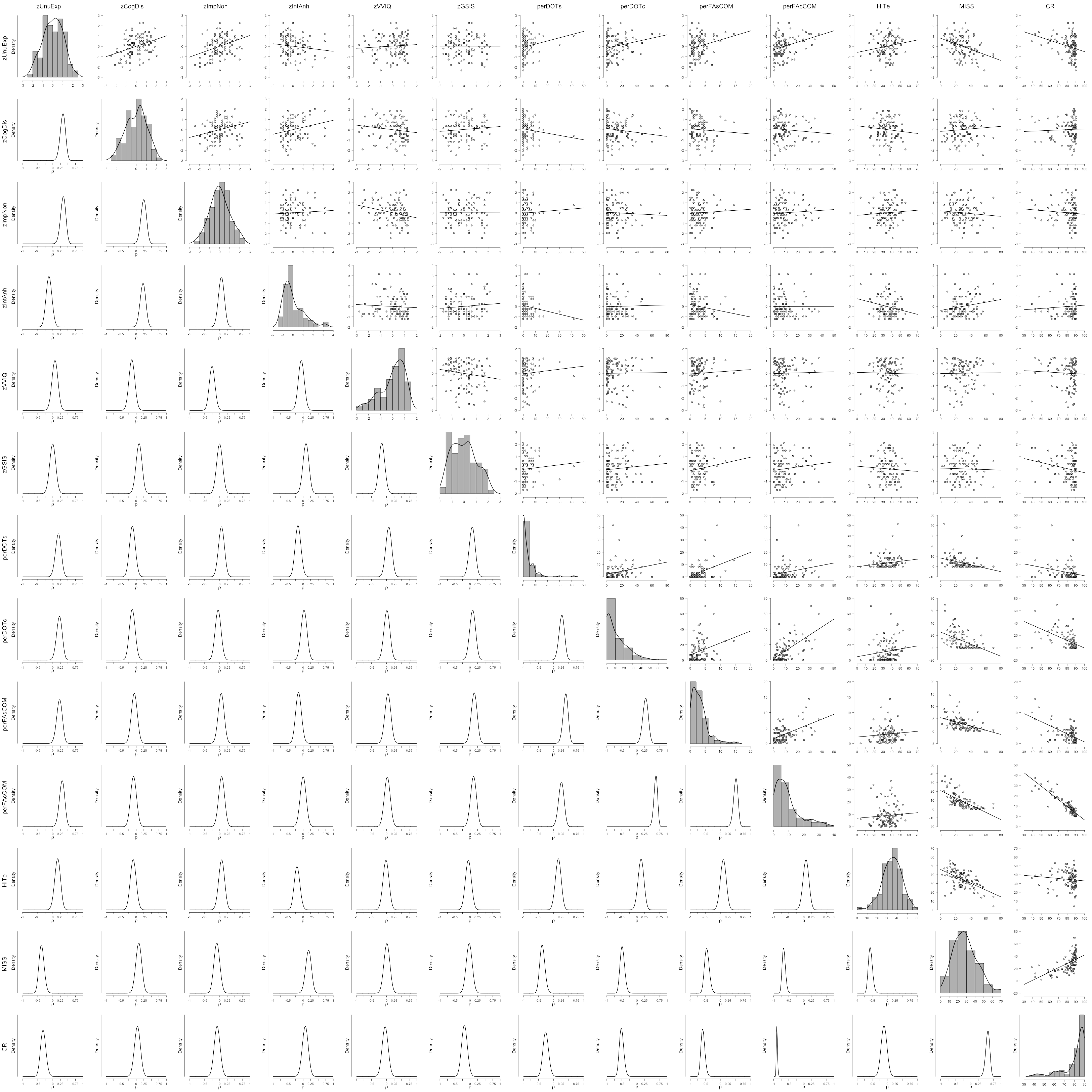
**

| **Experiment 1: Bayesian Linear Regression RDT:**   \| \| **Model Comparison - perDOTs** \| \| \| \| \| \| \| \| \| \| \| \| \| --- \| --- \| --- \| --- \| --- \| --- \| --- \| --- \| --- \| --- \| --- \| --- \| \| **Models** \| \| **P(M)** \| \| **P(M\|data)** \| \| **BF _M_** \| \| **BF _10_** \| \| **% error** \| \| \| Null model \|  \| 0.063 \|  \| 0.096 \|  \| 1.591 \|  \| 1.000 \|  \|  \|  \| \| UnEx \|  \| 0.063 \|  \| 0.104 \|  \| 1.741 \|  \| 1.085 \|  \| 0.008 \|  \| \| CogDis \|  \| 0.063 \|  \| 0.042 \|  \| 0.660 \|  \| 0.439 \|  \| 2.442e -5 \|  \| \| UnEx + CogDis \|  \| 0.063 \|  \| 0.211 \|  \| 4.006 \|  \| 2.198 \|  \| 5.097e -5 \|  \| \| ImpNon \|  \| 0.063 \|  \| 0.024 \|  \| 0.363 \|  \| 0.247 \|  \| 3.772e -5 \|  \| \| UnEx + ImpNon \|  \| 0.063 \|  \| 0.030 \|  \| 0.463 \|  \| 0.312 \|  \| 2.840e -5 \|  \| \| CogDis + ImpNon \|  \| 0.063 \|  \| 0.019 \|  \| 0.290 \|  \| 0.198 \|  \| 2.427e -5 \|  \| \| UnEx + CogDis + ImpNon \|  \| 0.063 \|  \| 0.070 \|  \| 1.137 \|  \| 0.735 \|  \| 1.921e -5 \|  \| \| IntAnh \|  \| 0.063 \|  \| 0.076 \|  \| 1.240 \|  \| 0.797 \|  \| 0.011 \|  \| \| UnEx + IntAnh \|  \| 0.063 \|  \| 0.082 \|  \| 1.335 \|  \| 0.852 \|  \| 3.897e -5 \|  \| \| CogDis + IntAnh \|  \| 0.063 \|  \| 0.032 \|  \| 0.502 \|  \| 0.338 \|  \| 2.915e -5 \|  \| \| UnEx + CogDis + IntAnh \|  \| 0.063 \|  \| 0.102 \|  \| 1.705 \|  \| 1.065 \|  \| 1.238e -5 \|  \| \| ImpNon + IntAnh \|  \| 0.063 \|  \| 0.028 \|  \| 0.429 \|  \| 0.290 \|  \| 2.772e -5 \|  \| \| UnEx + ImpNon + IntAnh \|  \| 0.063 \|  \| 0.028 \|  \| 0.427 \|  \| 0.289 \|  \| 4.957e -5 \|  \| \| CogDis + ImpNon + IntAnh \|  \| 0.063 \|  \| 0.017 \|  \| 0.259 \|  \| 0.177 \|  \| 7.559e -5 \|  \| \| UnEx + CogDis + ImpNon + IntAnh \|  \| 0.063 \|  \| 0.039 \|  \| 0.612 \|  \| 0.409 \|  \| 0.002 \|  \| \|  \| \| \| \| \| \| \| \| \| \| \| \|  \| **Analysis of Effects - perDOTs** \| \| \| \| \| \| \| \| \| \| \| \| \| \| \| \| \| --- \| --- \| --- \| --- \| --- \| --- \| --- \| --- \| --- \| --- \| --- \| --- \| --- \| --- \| --- \| --- \| \| **Effects** \| \| **P(incl)** \| \| **P(incl\|data)** \| \| **BF _Inclusion_** \| \| **BF _Backward_** \| \| **% errorB** \| \| **BF _Forward_** \| \| **% errorF** \| \| \| UnEx \|  \| 0.500 \|  \| 0.666 \|  \| 1.992 \|  \| 2.306 \|  \| 0.002 \|  \| 1.085 \|  \| 0.008 \|  \| \| CogDis \|  \| 0.500 \|  \| 0.533 \|  \| 1.141 \|  \| 1.415 \|  \| 0.002 \|  \| 0.439 \|  \| 2.442e -5 \|  \| \| ImpNon \|  \| 0.500 \|  \| 0.255 \|  \| 0.342 \|  \| 0.384 \|  \| 0.002 \|  \| 0.247 \|  \| 3.772e -5 \|  \| \| IntAnh \|  \| 0.500 \|  \| 0.404 \|  \| 0.678 \|  \| 0.556 \|  \| 0.002 \|  \| 0.797 \|  \| 0.011 \|  \| \|  \| \| \| \| \| \| \| \| \| \| \| \| \| \| \| \|  \| **Model Comparison - perDOTc** \| \| \| \| \| \| \| \| \| \| \| \| \| --- \| --- \| --- \| --- \| --- \| --- \| --- \| --- \| --- \| --- \| --- \| --- \| \| **Models** \| \| **P(M)** \| \| **P(M\|data)** \| \| **BF _M_** \| \| **BF _10_** \| \| **% error** \| \| \| Null model \|  \| 0.063 \|  \| 0.040 \|  \| 0.629 \|  \| 1.000 \|  \|  \|  \| \| UnEx \|  \| 0.063 \|  \| 0.087 \|  \| 1.435 \|  \| 2.171 \|  \| 0.004 \|  \| \| CogDis \|  \| 0.063 \|  \| 0.019 \|  \| 0.284 \|  \| 0.463 \|  \| 2.343e -5 \|  \| \| UnEx + CogDis \|  \| 0.063 \|  \| 0.258 \|  \| 5.225 \|  \| 6.423 \|  \| 6.709e -5 \|  \| \| ImpNon \|  \| 0.063 \|  \| 0.010 \|  \| 0.144 \|  \| 0.236 \|  \| 3.889e -5 \|  \| \| UnEx + ImpNon \|  \| 0.063 \|  \| 0.061 \|  \| 0.976 \|  \| 1.519 \|  \| 4.604e -5 \|  \| \| CogDis + ImpNon \|  \| 0.063 \|  \| 0.006 \|  \| 0.085 \|  \| 0.140 \|  \| 2.145e -5 \|  \| \| UnEx + CogDis + ImpNon \|  \| 0.063 \|  \| 0.142 \|  \| 2.477 \|  \| 3.523 \|  \| 0.005 \|  \| \| IntAnh \|  \| 0.063 \|  \| 0.009 \|  \| 0.132 \|  \| 0.218 \|  \| 4.117e -5 \|  \| \| UnEx + IntAnh \|  \| 0.063 \|  \| 0.029 \|  \| 0.440 \|  \| 0.709 \|  \| 3.688e -5 \|  \| \| CogDis + IntAnh \|  \| 0.063 \|  \| 0.007 \|  \| 0.099 \|  \| 0.164 \|  \| 2.267e -5 \|  \| \| UnEx + CogDis + IntAnh \|  \| 0.063 \|  \| 0.180 \|  \| 3.297 \|  \| 4.479 \|  \| 0.006 \|  \| \| ImpNon + IntAnh \|  \| 0.063 \|  \| 0.003 \|  \| 0.046 \|  \| 0.076 \|  \| 1.693e -5 \|  \| \| UnEx + ImpNon + IntAnh \|  \| 0.063 \|  \| 0.026 \|  \| 0.401 \|  \| 0.647 \|  \| 2.212e -5 \|  \| \| CogDis + ImpNon + IntAnh \|  \| 0.063 \|  \| 0.002 \|  \| 0.036 \|  \| 0.060 \|  \| 1.645e -4 \|  \| \| UnEx + CogDis + ImpNon + IntAnh \|  \| 0.063 \|  \| 0.122 \|  \| 2.084 \|  \| 3.033 \|  \| 0.003 \|  \| \|  \| \| \| \| \| \| \| \| \| \| \| \|  \| **Analysis of Effects - perDOTc** \| \| \| \| \| \| \| \| \| \| \| \| \| \| \| \| \| --- \| --- \| --- \| --- \| --- \| --- \| --- \| --- \| --- \| --- \| --- \| --- \| --- \| --- \| --- \| --- \| \| **Effects** \| \| **P(incl)** \| \| **P(incl\|data)** \| \| **BF _Inclusion_** \| \| **BF _Backward_** \| \| **% errorB** \| \| **BF _Forward_** \| \| **% errorF** \| \| \| UnEx \|  \| 0.500 \|  \| 0.905 \|  \| 9.547 \|  \| 50.522 \|  \| 0.003 \|  \| 2.171 \|  \| 0.004 \|  \| \| CogDis \|  \| 0.500 \|  \| 0.735 \|  \| 2.780 \|  \| 4.685 \|  \| 0.003 \|  \| 0.463 \|  \| 2.343e -5 \|  \| \| ImpNon \|  \| 0.500 \|  \| 0.371 \|  \| 0.591 \|  \| 0.677 \|  \| 0.007 \|  \| 0.236 \|  \| 3.889e -5 \|  \| \| IntAnh \|  \| 0.500 \|  \| 0.378 \|  \| 0.607 \|  \| 0.861 \|  \| 0.006 \|  \| 0.218 \|  \| 4.117e -5 \|  \| \|  \| \| \| \| \| \| \| \| \| \| \| \| \| \| \| \|   **Model Comparison - perFAsCOM** \| \| \| \| \| \| \| \| \| \| \| \| \| --- \| --- \| --- \| --- \| --- \| --- \| --- \| --- \| --- \| --- \| --- \| --- \| --- \| --- \| --- \| --- \| --- \| --- \| --- \| --- \| --- \| --- \| --- \| --- \| --- \| --- \| --- \| --- \| --- \| --- \| --- \| --- \| --- \| --- \| --- \| --- \| --- \| --- \| --- \| --- \| --- \| --- \| --- \| --- \| --- \| --- \| --- \| --- \| --- \| --- \| --- \| --- \| --- \| --- \| --- \| --- \| --- \| --- \| --- \| --- \| --- \| --- \| --- \| --- \| --- \| --- \| --- \| --- \| --- \| --- \| --- \| --- \| --- \| --- \| --- \| --- \| --- \| --- \| --- \| --- \| --- \| --- \| --- \| --- \| --- \| --- \| --- \| --- \| --- \| --- \| --- \| --- \| --- \| --- \| --- \| --- \| --- \| --- \| --- \| --- \| --- \| --- \| --- \| --- \| --- \| --- \| --- \| --- \| --- \| --- \| --- \| --- \| --- \| --- \| --- \| --- \| --- \| --- \| --- \| --- \| --- \| --- \| --- \| --- \| --- \| --- \| --- \| --- \| --- \| --- \| --- \| --- \| --- \| --- \| --- \| --- \| --- \| --- \| --- \| --- \| --- \| --- \| --- \| --- \| --- \| --- \| --- \| --- \| --- \| --- \| --- \| --- \| --- \| --- \| --- \| --- \| --- \| --- \| --- \| --- \| --- \| --- \| --- \| --- \| --- \| --- \| --- \| --- \| --- \| --- \| --- \| --- \| --- \| --- \| --- \| --- \| --- \| --- \| --- \| --- \| --- \| --- \| --- \| --- \| --- \| --- \| --- \| --- \| --- \| --- \| --- \| --- \| --- \| --- \| --- \| --- \| --- \| --- \| --- \| --- \| --- \| --- \| --- \| --- \| --- \| --- \| --- \| --- \| --- \| --- \| --- \| --- \| --- \| --- \| --- \| --- \| --- \| --- \| --- \| --- \| --- \| --- \| --- \| --- \| --- \| --- \| --- \| --- \| --- \| --- \| --- \| --- \| --- \| --- \| --- \| --- \| --- \| --- \| --- \| --- \| --- \| --- \| --- \| --- \| --- \| --- \| --- \| --- \| --- \| --- \| --- \| --- \| --- \| --- \| --- \| --- \| --- \| --- \| --- \| --- \| --- \| --- \| --- \| --- \| --- \| --- \| --- \| --- \| --- \| --- \| --- \| --- \| --- \| --- \| --- \| --- \| --- \| --- \| --- \| --- \| --- \| --- \| --- \| --- \| --- \| --- \| --- \| --- \| --- \| --- \| --- \| --- \| --- \| --- \| --- \| --- \| --- \| --- \| --- \| --- \| --- \| --- \| --- \| --- \| --- \| --- \| --- \| --- \| --- \| --- \| --- \| --- \| --- \| --- \| --- \| --- \| --- \| --- \| --- \| --- \| --- \| --- \| --- \| --- \| --- \| --- \| --- \| --- \| --- \| --- \| --- \| --- \| --- \| --- \| --- \| --- \| --- \| --- \| --- \| --- \| --- \| --- \| --- \| --- \| --- \| --- \| --- \| --- \| --- \| --- \| --- \| --- \| --- \| --- \| --- \| --- \| --- \| --- \| --- \| --- \| --- \| --- \| --- \| --- \| --- \| --- \| --- \| --- \| --- \| --- \| --- \| --- \| --- \| --- \| --- \| --- \| --- \| --- \| --- \| --- \| --- \| --- \| --- \| --- \| --- \| --- \| --- \| --- \| --- \| --- \| --- \| --- \| --- \| --- \| --- \| --- \| --- \| --- \| --- \| --- \| --- \| --- \| --- \| --- \| --- \| --- \| --- \| --- \| --- \| --- \| --- \| --- \| --- \| --- \| --- \| --- \| --- \| --- \| --- \| --- \| --- \| --- \| --- \| --- \| --- \| --- \| --- \| --- \| --- \| --- \| --- \| --- \| --- \| --- \| --- \| --- \| --- \| --- \| --- \| --- \| --- \| --- \| --- \| --- \| --- \| --- \| --- \| --- \| --- \| --- \| --- \| --- \| --- \| --- \| --- \| --- \| --- \| --- \| --- \| --- \| --- \| --- \| --- \| --- \| --- \| --- \| --- \| --- \| --- \| --- \| --- \| --- \| --- \| --- \| --- \| --- \| --- \| --- \| --- \| --- \| --- \| --- \| --- \| --- \| --- \| --- \| --- \| --- \| --- \| --- \| --- \| --- \| --- \| --- \| --- \| --- \| --- \| --- \| --- \| --- \| --- \| --- \| --- \| --- \| --- \| --- \| --- \| --- \| --- \| --- \| --- \| --- \| --- \| --- \| --- \| --- \| --- \| --- \| --- \| --- \| --- \| --- \| --- \| --- \| --- \| --- \| --- \| --- \| --- \| --- \| --- \| --- \| --- \| --- \| --- \| --- \| --- \| --- \| --- \| --- \| --- \| --- \| --- \| --- \| --- \| --- \| --- \| --- \| --- \| --- \| --- \| --- \| --- \| --- \| --- \| --- \| --- \| --- \| --- \| --- \| --- \| --- \| --- \| --- \| --- \| --- \| --- \| --- \| --- \| --- \| --- \| --- \| --- \| --- \| --- \| --- \| --- \| --- \| --- \| --- \| --- \| --- \| --- \| --- \| --- \| --- \| --- \| --- \| --- \| --- \| --- \| --- \| --- \| --- \| --- \| --- \| --- \| --- \| --- \| --- \| --- \| --- \| --- \| --- \| --- \| --- \| --- \| --- \| --- \| --- \| --- \| --- \| --- \| --- \| --- \| --- \| --- \| --- \| --- \| --- \| --- \| --- \| --- \| --- \| --- \| --- \| --- \| --- \| --- \| --- \| --- \| --- \| --- \| --- \| --- \| --- \| --- \| --- \| --- \| --- \| --- \| --- \| --- \| --- \| --- \| --- \| --- \| --- \| --- \| --- \| --- \| --- \| --- \| --- \| --- \| --- \| --- \| --- \| --- \| --- \| --- \| --- \| --- \| --- \| --- \| --- \| --- \| --- \| --- \| --- \| --- \| --- \| --- \| --- \| --- \| --- \| --- \| --- \| --- \| --- \| --- \| --- \| --- \| --- \| --- \| --- \| --- \| --- \| --- \| --- \| --- \| --- \| \| **Models** \| \| **P(M)** \| \| **P(M\|data)** \| \| **BF _M_** \| \| **BF _10_** \| \| **% error** \| \| \| Null model \|  \| 0.063 \|  \| 0.089 \|  \| 1.463 \|  \| 1.000 \|  \|  \|  \| \| UnEx \|  \| 0.063 \|  \| 0.209 \|  \| 3.964 \|  \| 2.352 \|  \| 0.004 \|  \| \| CogDis \|  \| 0.063 \|  \| 0.022 \|  \| 0.341 \|  \| 0.250 \|  \| 3.733e -5 \|  \| \| UnEx + CogDis \|  \| 0.063 \|  \| 0.168 \|  \| 3.020 \|  \| 1.886 \|  \| 4.889e -5 \|  \| \| ImpNon \|  \| 0.063 \|  \| 0.021 \|  \| 0.321 \|  \| 0.236 \|  \| 3.897e -5 \|  \| \| UnEx + ImpNon \|  \| 0.063 \|  \| 0.061 \|  \| 0.967 \|  \| 0.681 \|  \| 3.644e -5 \|  \| \| CogDis + ImpNon \|  \| 0.063 \|  \| 0.008 \|  \| 0.128 \|  \| 0.095 \|  \| 1.847e -5 \|  \| \| UnEx + CogDis + ImpNon \|  \| 0.063 \|  \| 0.055 \|  \| 0.873 \|  \| 0.619 \|  \| 2.324e -5 \|  \| \| IntAnh \|  \| 0.063 \|  \| 0.055 \|  \| 0.871 \|  \| 0.618 \|  \| 1.844e -5 \|  \| \| UnEx + IntAnh \|  \| 0.063 \|  \| 0.122 \|  \| 2.088 \|  \| 1.375 \|  \| 4.477e -5 \|  \| \| CogDis + IntAnh \|  \| 0.063 \|  \| 0.017 \|  \| 0.256 \|  \| 0.189 \|  \| 2.388e -5 \|  \| \| UnEx + CogDis + IntAnh \|  \| 0.063 \|  \| 0.077 \|  \| 1.257 \|  \| 0.870 \|  \| 1.580e -5 \|  \| \| ImpNon + IntAnh \|  \| 0.063 \|  \| 0.019 \|  \| 0.291 \|  \| 0.214 \|  \| 2.497e -5 \|  \| \| UnEx + ImpNon + IntAnh \|  \| 0.063 \|  \| 0.041 \|  \| 0.641 \|  \| 0.461 \|  \| 3.161e -5 \|  \| \| CogDis + ImpNon + IntAnh \|  \| 0.063 \|  \| 0.007 \|  \| 0.112 \|  \| 0.084 \|  \| 1.327e -4 \|  \| \| UnEx + CogDis + ImpNon + IntAnh \|  \| 0.063 \|  \| 0.029 \|  \| 0.442 \|  \| 0.322 \|  \| 0.002 \|  \| \|  \| \| \| \| \| \| \| \| \| \| \| \|  \| **Analysis of Effects - perFAsCOM** \| \| \| \| \| \| \| \| \| \| \| \| \| \| \| \| \| --- \| --- \| --- \| --- \| --- \| --- \| --- \| --- \| --- \| --- \| --- \| --- \| --- \| --- \| --- \| --- \| \| **Effects** \| \| **P(incl)** \| \| **P(incl\|data)** \| \| **BF _Inclusion_** \| \| **BF _Backward_** \| \| **% errorB** \| \| **BF _Forward_** \| \| **% errorF** \| \| \| UnEx \|  \| 0.500 \|  \| 0.761 \|  \| 3.190 \|  \| 3.857 \|  \| 0.002 \|  \| 2.352 \|  \| 0.004 \|  \| \| CogDis \|  \| 0.500 \|  \| 0.383 \|  \| 0.622 \|  \| 0.699 \|  \| 0.002 \|  \| 0.250 \|  \| 3.733e -5 \|  \| \| ImpNon \|  \| 0.500 \|  \| 0.241 \|  \| 0.318 \|  \| 0.370 \|  \| 0.002 \|  \| 0.236 \|  \| 3.897e -5 \|  \| \| IntAnh \|  \| 0.500 \|  \| 0.367 \|  \| 0.581 \|  \| 0.521 \|  \| 0.002 \|  \| 0.618 \|  \| 1.844e -5 \|  \| \|  \| \| \| \| \| \| \| \| \| \| \| \| \| \| \| \|  \| **Model Comparison - perFAsCOM** \| \| \| \| \| \| \| \| \| \| \| \| \| --- \| --- \| --- \| --- \| --- \| --- \| --- \| --- \| --- \| --- \| --- \| --- \| \| **Models** \| \| **P(M)** \| \| **P(M\|data)** \| \| **BF _M_** \| \| **BF _10_** \| \| **% error** \| \| \| Null model \|  \| 0.063 \|  \| 0.089 \|  \| 1.463 \|  \| 1.000 \|  \|  \|  \| \| UnEx \|  \| 0.063 \|  \| 0.209 \|  \| 3.964 \|  \| 2.352 \|  \| 0.004 \|  \| \| CogDis \|  \| 0.063 \|  \| 0.022 \|  \| 0.341 \|  \| 0.250 \|  \| 3.733e -5 \|  \| \| UnEx + CogDis \|  \| 0.063 \|  \| 0.168 \|  \| 3.020 \|  \| 1.886 \|  \| 4.889e -5 \|  \| \| ImpNon \|  \| 0.063 \|  \| 0.021 \|  \| 0.321 \|  \| 0.236 \|  \| 3.897e -5 \|  \| \| UnEx + ImpNon \|  \| 0.063 \|  \| 0.061 \|  \| 0.967 \|  \| 0.681 \|  \| 3.644e -5 \|  \| \| CogDis + ImpNon \|  \| 0.063 \|  \| 0.008 \|  \| 0.128 \|  \| 0.095 \|  \| 1.847e -5 \|  \| \| UnEx + CogDis + ImpNon \|  \| 0.063 \|  \| 0.055 \|  \| 0.873 \|  \| 0.619 \|  \| 2.324e -5 \|  \| \| IntAnh \|  \| 0.063 \|  \| 0.055 \|  \| 0.871 \|  \| 0.618 \|  \| 1.844e -5 \|  \| \| UnEx + IntAnh \|  \| 0.063 \|  \| 0.122 \|  \| 2.088 \|  \| 1.375 \|  \| 4.477e -5 \|  \| \| CogDis + IntAnh \|  \| 0.063 \|  \| 0.017 \|  \| 0.256 \|  \| 0.189 \|  \| 2.388e -5 \|  \| \| UnEx + CogDis + IntAnh \|  \| 0.063 \|  \| 0.077 \|  \| 1.257 \|  \| 0.870 \|  \| 1.580e -5 \|  \| \| ImpNon + IntAnh \|  \| 0.063 \|  \| 0.019 \|  \| 0.291 \|  \| 0.214 \|  \| 2.497e -5 \|  \| \| UnEx + ImpNon + IntAnh \|  \| 0.063 \|  \| 0.041 \|  \| 0.641 \|  \| 0.461 \|  \| 3.161e -5 \|  \| \| CogDis + ImpNon + IntAnh \|  \| 0.063 \|  \| 0.007 \|  \| 0.112 \|  \| 0.084 \|  \| 1.327e -4 \|  \| \| UnEx + CogDis + ImpNon + IntAnh \|  \| 0.063 \|  \| 0.029 \|  \| 0.442 \|  \| 0.322 \|  \| 0.002 \|  \| \|  \| \| \| \| \| \| \| \| \| \| \| \|   **Model Comparison - perFAcCOM** |
| --- | --- | --- | --- | --- | --- | --- | --- | --- | --- | --- | --- | --- | --- | --- | --- | --- | --- | --- | --- | --- | --- | --- | --- | --- | --- | --- | --- | --- | --- | --- | --- | --- | --- | --- | --- | --- | --- | --- | --- | --- | --- | --- | --- | --- | --- | --- | --- | --- | --- | --- | --- | --- | --- | --- | --- | --- | --- | --- | --- | --- | --- | --- | --- | --- | --- | --- | --- | --- | --- | --- | --- | --- | --- | --- | --- | --- | --- | --- | --- | --- | --- | --- | --- | --- | --- | --- | --- | --- | --- | --- | --- | --- | --- | --- | --- | --- | --- | --- | --- | --- | --- | --- | --- | --- | --- | --- | --- | --- | --- | --- | --- | --- | --- | --- | --- | --- | --- | --- | --- | --- | --- | --- | --- | --- | --- | --- | --- | --- | --- | --- | --- | --- | --- | --- | --- | --- | --- | --- | --- | --- | --- | --- | --- | --- | --- | --- | --- | --- | --- | --- | --- | --- | --- | --- | --- | --- | --- | --- | --- | --- | --- | --- | --- | --- | --- | --- | --- | --- | --- | --- | --- | --- | --- | --- | --- | --- | --- | --- | --- | --- | --- | --- | --- | --- | --- | --- | --- | --- | --- | --- | --- | --- | --- | --- | --- | --- | --- | --- | --- | --- | --- | --- | --- | --- | --- | --- | --- | --- | --- | --- | --- | --- | --- | --- | --- | --- | --- | --- | --- | --- | --- | --- | --- | --- | --- | --- | --- | --- | --- | --- | --- | --- | --- | --- | --- | --- | --- | --- | --- | --- | --- | --- | --- | --- | --- | --- | --- | --- | --- | --- | --- | --- | --- | --- | --- | --- | --- | --- | --- | --- | --- | --- | --- | --- | --- | --- | --- | --- | --- | --- | --- | --- | --- | --- | --- | --- | --- | --- | --- | --- | --- | --- | --- | --- | --- | --- | --- | --- | --- | --- | --- | --- | --- | --- | --- | --- | --- | --- | --- | --- | --- | --- | --- | --- | --- | --- | --- | --- | --- | --- | --- | --- | --- | --- | --- | --- | --- | --- | --- | --- | --- | --- | --- | --- | --- | --- | --- | --- | --- | --- | --- | --- | --- | --- | --- | --- | --- | --- | --- | --- | --- | --- | --- | --- | --- | --- | --- | --- | --- | --- | --- | --- | --- | --- | --- | --- | --- | --- | --- | --- | --- | --- | --- | --- | --- | --- | --- | --- | --- | --- | --- | --- | --- | --- | --- | --- | --- | --- | --- | --- | --- | --- | --- | --- | --- | --- | --- | --- | --- | --- | --- | --- | --- | --- | --- | --- | --- | --- | --- | --- | --- | --- | --- | --- | --- | --- | --- | --- | --- | --- | --- | --- | --- | --- | --- | --- | --- | --- | --- | --- | --- | --- | --- | --- | --- | --- | --- | --- | --- | --- | --- | --- | --- | --- | --- | --- | --- | --- | --- | --- | --- | --- | --- | --- | --- | --- | --- | --- | --- | --- | --- | --- | --- | --- | --- | --- | --- | --- | --- | --- | --- | --- | --- | --- | --- | --- | --- | --- | --- | --- | --- | --- | --- | --- | --- | --- | --- | --- | --- | --- | --- | --- | --- | --- | --- | --- | --- | --- | --- | --- | --- | --- | --- | --- | --- | --- | --- | --- | --- | --- | --- | --- | --- | --- | --- | --- | --- | --- | --- | --- | --- | --- | --- | --- | --- | --- | --- | --- | --- | --- | --- | --- | --- | --- | --- | --- | --- | --- | --- | --- | --- | --- | --- | --- | --- | --- | --- | --- | --- | --- | --- | --- | --- | --- | --- | --- | --- | --- | --- | --- | --- | --- | --- | --- | --- | --- | --- | --- | --- | --- | --- | --- | --- | --- | --- | --- | --- | --- | --- | --- | --- | --- | --- | --- | --- | --- | --- | --- | --- | --- | --- | --- | --- | --- | --- | --- | --- | --- | --- | --- | --- | --- | --- | --- | --- | --- | --- | --- | --- | --- | --- | --- | --- | --- | --- | --- | --- | --- | --- | --- | --- | --- | --- | --- | --- | --- | --- | --- | --- | --- | --- | --- | --- | --- | --- | --- | --- | --- | --- | --- | --- | --- | --- | --- | --- | --- | --- | --- | --- | --- | --- | --- | --- | --- | --- | --- | --- | --- | --- | --- | --- | --- | --- | --- | --- | --- | --- | --- | --- | --- | --- | --- | --- | --- | --- | --- | --- | --- | --- | --- | --- | --- | --- | --- | --- | --- | --- | --- | --- | --- | --- | --- | --- | --- | --- | --- | --- | --- | --- | --- | --- | --- | --- | --- | --- | --- | --- | --- | --- | --- | --- | --- | --- | --- | --- | --- | --- | --- | --- | --- | --- | --- | --- | --- | --- | --- | --- | --- | --- | --- | --- | --- | --- | --- | --- | --- | --- | --- | --- | --- | --- | --- | --- | --- | --- | --- | --- | --- | --- | --- | --- | --- | --- | --- | --- | --- | --- | --- | --- | --- | --- | --- | --- | --- | --- | --- | --- | --- | --- | --- | --- | --- | --- | --- | --- | --- | --- | --- | --- | --- | --- | --- | --- | --- | --- | --- | --- | --- | --- | --- | --- | --- | --- | --- | --- | --- | --- | --- | --- | --- | --- | --- | --- | --- | --- | --- | --- | --- | --- | --- | --- | --- | --- | --- | --- | --- | --- | --- | --- | --- | --- | --- | --- | --- | --- | --- | --- | --- | --- | --- | --- | --- | --- | --- | --- | --- | --- | --- | --- | --- | --- | --- | --- | --- | --- | --- | --- | --- | --- | --- | --- | --- | --- | --- | --- | --- | --- | --- | --- | --- | --- | --- | --- | --- | --- | --- | --- | --- | --- | --- | --- | --- | --- | --- | --- | --- | --- | --- | --- | --- | --- | --- | --- | --- | --- | --- | --- | --- | --- | --- | --- | --- | --- | --- | --- | --- | --- | --- | --- | --- | --- | --- | --- | --- | --- | --- | --- | --- | --- | --- | --- | --- | --- | --- | --- | --- | --- | --- | --- | --- | --- | --- | --- | --- | --- | --- | --- | --- | --- | --- | --- | --- | --- | --- | --- | --- | --- | --- | --- | --- | --- | --- | --- | --- | --- | --- | --- | --- | --- | --- | --- | --- | --- | --- | --- | --- | --- | --- | --- | --- | --- | --- | --- | --- | --- | --- | --- | --- | --- | --- | --- | --- | --- | --- | --- | --- | --- | --- | --- | --- | --- | --- | --- | --- | --- | --- | --- | --- | --- | --- | --- | --- | --- | --- | --- | --- | --- | --- | --- | --- | --- | --- | --- | --- | --- | --- | --- | --- | --- | --- | --- | --- | --- | --- | --- | --- | --- | --- | --- | --- | --- | --- | --- | --- | --- | --- | --- | --- | --- | --- | --- | --- | --- | --- | --- | --- | --- | --- | --- | --- | --- | --- | --- | --- | --- | --- | --- | --- | --- | --- | --- | --- | --- | --- | --- | --- | --- | --- | --- | --- | --- | --- | --- | --- | --- | --- | --- | --- | --- | --- | --- | --- | --- | --- | --- | --- | --- | --- | --- | --- | --- | --- | --- | --- | --- | --- | --- | --- | --- | --- | --- | --- | --- | --- | --- | --- | --- | --- | --- | --- | --- | --- | --- | --- | --- | --- | --- | --- | --- | --- | --- | --- | --- | --- | --- | --- | --- | --- | --- | --- | --- | --- | --- | --- | --- | --- | --- | --- | --- | --- | --- | --- | --- | --- | --- | --- | --- | --- | --- | --- | --- | --- | --- | --- | --- | --- | --- | --- | --- | --- | --- | --- | --- | --- | --- | --- | --- | --- | --- | --- | --- | --- | --- | --- | --- | --- | --- | --- | --- | --- | --- | --- | --- | --- | --- | --- | --- | --- | --- | --- | --- | --- | --- | --- | --- | --- | --- | --- | --- | --- | --- | --- | --- | --- | --- | --- | --- | --- | --- | --- | --- | --- | --- | --- | --- | --- | --- | --- | --- | --- | --- | --- | --- | --- | --- | --- | --- | --- | --- | --- | --- | --- | --- | --- | --- | --- | --- | --- | --- | --- | --- | --- | --- | --- | --- | --- | --- | --- | --- | --- | --- | --- | --- | --- | --- | --- | --- | --- | --- | --- | --- | --- | --- | --- | --- | --- | --- | --- |

**Experiment 1: Bayesian Linear Regression POM:**

| **Model Comparison - perFAsCOM** | | | | | | | | | | | |
| --- | --- | --- | --- | --- | --- | --- | --- | --- | --- | --- | --- |
| **Models** | | **P(M)** | | **P(M\|data)** | | **BF _M_** | | **BF _10_** | | **% error** | |
| Null model |  | 0.063 |  | 0.089 |  | 1.463 |  | 1.000 |  |  |  |
| UnEx |  | 0.063 |  | 0.209 |  | 3.964 |  | 2.352 |  | 0.004 |  |
| CogDis |  | 0.063 |  | 0.022 |  | 0.341 |  | 0.250 |  | 3.733e -5 |  |
| UnEx + CogDis |  | 0.063 |  | 0.168 |  | 3.020 |  | 1.886 |  | 4.889e -5 |  |
| ImpNon |  | 0.063 |  | 0.021 |  | 0.321 |  | 0.236 |  | 3.897e -5 |  |
| UnEx + ImpNon |  | 0.063 |  | 0.061 |  | 0.967 |  | 0.681 |  | 3.644e -5 |  |
| CogDis + ImpNon |  | 0.063 |  | 0.008 |  | 0.128 |  | 0.095 |  | 1.847e -5 |  |
| UnEx + CogDis + ImpNon |  | 0.063 |  | 0.055 |  | 0.873 |  | 0.619 |  | 2.324e -5 |  |
| IntAnh |  | 0.063 |  | 0.055 |  | 0.871 |  | 0.618 |  | 1.844e -5 |  |
| UnEx + IntAnh |  | 0.063 |  | 0.122 |  | 2.088 |  | 1.375 |  | 4.477e -5 |  |
| CogDis + IntAnh |  | 0.063 |  | 0.017 |  | 0.256 |  | 0.189 |  | 2.388e -5 |  |
| UnEx + CogDis + IntAnh |  | 0.063 |  | 0.077 |  | 1.257 |  | 0.870 |  | 1.580e -5 |  |
| ImpNon + IntAnh |  | 0.063 |  | 0.019 |  | 0.291 |  | 0.214 |  | 2.497e -5 |  |
| UnEx + ImpNon + IntAnh |  | 0.063 |  | 0.041 |  | 0.641 |  | 0.461 |  | 3.161e -5 |  |
| CogDis + ImpNon + IntAnh |  | 0.063 |  | 0.007 |  | 0.112 |  | 0.084 |  | 1.327e -4 |  |
| UnEx + CogDis + ImpNon + IntAnh |  | 0.063 |  | 0.029 |  | 0.442 |  | 0.322 |  | 0.002 |  |
|  | | | | | | | | | | | |

| **Analysis of Effects - perFAsCOM** | | | | | | | | | | | | | | | |
| --- | --- | --- | --- | --- | --- | --- | --- | --- | --- | --- | --- | --- | --- | --- | --- |
| **Effects** | | **P(incl)** | | **P(incl\|data)** | | **BF _Inclusion_** | | **BF _Backward_** | | **% errorB** | | **BF _Forward_** | | **% errorF** | |
| UnEx |  | 0.500 |  | 0.761 |  | 3.190 |  | 3.857 |  | 0.002 |  | 2.352 |  | 0.004 |  |
| CogDis |  | 0.500 |  | 0.383 |  | 0.622 |  | 0.699 |  | 0.002 |  | 0.250 |  | 3.733e -5 |  |
| ImpNon |  | 0.500 |  | 0.241 |  | 0.318 |  | 0.370 |  | 0.002 |  | 0.236 |  | 3.897e -5 |  |
| IntAnh |  | 0.500 |  | 0.367 |  | 0.581 |  | 0.521 |  | 0.002 |  | 0.618 |  | 1.844e -5 |  |
|  | | | | | | | | | | | | | | | |

| **Model Comparison - perFAcCOM** | | | | | | | | | | | |
| --- | --- | --- | --- | --- | --- | --- | --- | --- | --- | --- | --- |
| **Models** | | **P(M)** | | **P(M\|data)** | | **BF _M_** | | **BF _10_** | | **% error** | |
| Null model |  | 0.063 |  | 0.006 |  | 0.097 |  | 1.000 |  |  |  |
| UnEx |  | 0.063 |  | 0.156 |  | 2.774 |  | 24.199 |  | 3.132e -5 |  |
| CogDis |  | 0.063 |  | 0.002 |  | 0.029 |  | 0.299 |  | 3.281e -5 |  |
| UnEx + CogDis |  | 0.063 |  | 0.368 |  | 8.716 |  | 56.987 |  | 1.090e -4 |  |
| ImpNon |  | 0.063 |  | 0.002 |  | 0.025 |  | 0.254 |  | 3.698e -5 |  |
| UnEx + ImpNon |  | 0.063 |  | 0.044 |  | 0.691 |  | 6.830 |  | 6.810e -5 |  |
| CogDis + ImpNon |  | 0.063 |  | 8.433e -4 |  | 0.013 |  | 0.131 |  | 2.088e -5 |  |
| UnEx + CogDis + ImpNon |  | 0.063 |  | 0.107 |  | 1.806 |  | 16.662 |  | 7.590e -7 |  |
| IntAnh |  | 0.063 |  | 0.001 |  | 0.020 |  | 0.209 |  | 4.233e -5 |  |
| UnEx + IntAnh |  | 0.063 |  | 0.043 |  | 0.668 |  | 6.612 |  | 6.757e -5 |  |
| CogDis + IntAnh |  | 0.063 |  | 6.004e -4 |  | 0.009 |  | 0.093 |  | 1.833e -5 |  |
| UnEx + CogDis + IntAnh |  | 0.063 |  | 0.189 |  | 3.507 |  | 29.381 |  | 2.883e -6 |  |
| ImpNon + IntAnh |  | 0.063 |  | 5.027e -4 |  | 0.008 |  | 0.078 |  | 1.707e -5 |  |
| UnEx + ImpNon + IntAnh |  | 0.063 |  | 0.015 |  | 0.232 |  | 2.359 |  | 0.006 |  |
| CogDis + ImpNon + IntAnh |  | 0.063 |  | 3.138e -4 |  | 0.005 |  | 0.049 |  | 1.866e -4 |  |
| UnEx + CogDis + ImpNon + IntAnh |  | 0.063 |  | 0.064 |  | 1.025 |  | 9.922 |  | 0.004 |  |
|  | | | | | | | | | | | |

| **Analysis of Effects - perFAcCOM** | | | | | | | | | | | | | | | |
| --- | --- | --- | --- | --- | --- | --- | --- | --- | --- | --- | --- | --- | --- | --- | --- |
| **Effects** | | **P(incl)** | | **P(incl\|data)** | | **BF _Inclusion_** | | **BF _Backward_** | | **% errorB** | | **BF _Forward_** | | **% errorF** | |
| UnEx |  | 0.500 |  | 0.986 |  | 72.414 |  | 203.902 |  | 0.004 |  | 24.199 |  | 3.132e -5 |  |
| CogDis |  | 0.500 |  | 0.732 |  | 2.733 |  | 4.205 |  | 0.008 |  | 0.299 |  | 3.281e -5 |  |
| ImpNon |  | 0.500 |  | 0.234 |  | 0.305 |  | 0.338 |  | 0.004 |  | 0.254 |  | 3.698e -5 |  |
| IntAnh |  | 0.500 |  | 0.314 |  | 0.458 |  | 0.595 |  | 0.004 |  | 0.209 |  | 4.233e -5 |  |
|  | | | | | | | | | | | | | | | |

**Experiment 2: Bayesian Pearson Correlations**

|  | | | | | | | | | | | | | | | | | |
| --- | --- | --- | --- | --- | --- | --- | --- | --- | --- | --- | --- | --- | --- | --- | --- | --- | --- |
|  | |  | | **UnEx** | | **CogDis** | | **ImpNon** | | **d_prime** | | **invbeta** | | **Hr** | | **Fr** | |
| UnEx |  | Pearson's r |  | — |  | 0.478 | *** | 0.516 | *** | -0.287 |  | -0.291 |  | 0.155 |  | 0.298 |  |
|  |  | BF₁₀ |  | — |  | 17003 |  | 168772 |  | 6.483 |  | 7.116 |  | 0.388 |  | 8.755 |  |
| CogDis |  | Pearson's r |  |  |  | — |  | 0.452 | *** | -0.297 |  | -0.249 |  | 0.073 |  | 0.259 |  |
|  |  | BF₁₀ |  |  |  | — |  | 4258 |  | 8.722 |  | 2.381 |  | 0.164 |  | 3.039 |  |
| ImpNon |  | Pearson's r |  |  |  |  |  | — |  | -0.151 |  | -0.146 |  | 0.068 |  | 0.144 |  |
|  |  | BF₁₀ |  |  |  |  |  | — |  | 0.368 |  | 0.341 |  | 0.159 |  | 0.333 |  |
| d_prime |  | Pearson's r |  |  |  |  |  |  |  | — |  | 0.830 | *** | -0.290 |  | -0.864 | *** |
|  |  | BF₁₀ |  |  |  |  |  |  |  | — |  | 5.732e +46 |  | 6.980 |  | 5.771e  +63 |  |
| invbeta |  | Pearson's r |  |  |  |  |  |  |  |  |  | — |  | -0.736 | *** | -0.905 | *** |
|  |  | BF₁₀ |  |  |  |  |  |  |  |  |  | — |  | 5.605e +23 |  | 3.023e +100 |  |
| Hr |  | Pearson's r |  |  |  |  |  |  |  |  |  |  |  | — |  | 0.667 | *** |
|  |  | BF₁₀ |  |  |  |  |  |  |  |  |  |  |  | — |  | 3.739e  +15 |  |
| Fr |  | Pearson's r |  |  |  |  |  |  |  |  |  |  |  |  |  | — |  |
|  |  | BF₁₀ |  |  |  |  |  |  |  |  |  |  |  |  |  | — |  |
|  | | | | | | | | | | | | | | | | | |
| * BF₁₀ > 10, ** , BF₁₀ > 30, *** BF₁₀ > 100 | | | | | | | | | | | | | | | | | |

**Experiment 2: Bayesian Correlation plot**

**
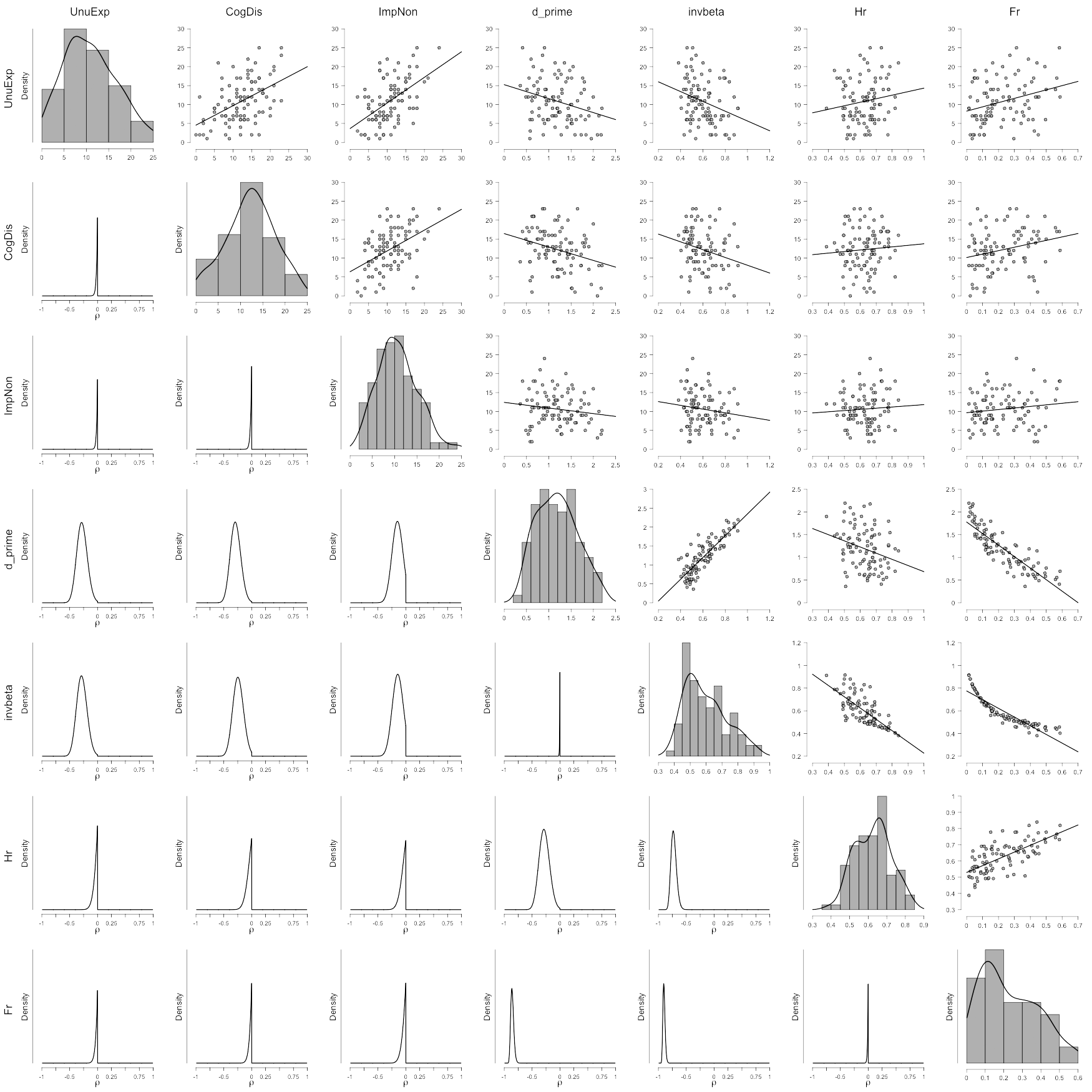
**

**Bibliography**

**1.** O'Keefe DJ (2003) Colloquy: Should familywise alpha be adjusted? Against familywise alpha adjustment. Human Communication Research 29: 431-447.

**2.** Love J, Selker, R., Marsman, M., Jamil, T., Dropmann, D., Verhagen, A. J., Ly, A., Gronau, Q. F., Smira, M., Epskamp, S., Matzke, D., Wild, A., Rouder, J. N., Morey, R. D. & Wagenmakers, E.-J. (2015) JASP. <https://jasp-stats.org/>.

**3.** Gelman A (2008) Objections to Bayesian statistics. Bayesian Analysis 3: 445-450.

**4.** Dienes Z (2014) Using Bayes to get the most out of non-significant results. Frontiers in psychology 5: 781.

**5.** Dienes Z (2011) Bayesian Versus Orthodox Statistics: Which Side Are You On? Perspect Psychol Sci 6: 274-290.

**6.** Kass RE and Raftery AE (1995) Bayes Factors. Journal of the American Statistical Association 90: 773-795.
